# RegAB Homolog of *Burkholderia pseudomallei* is the Master Regulator of Redox Control and involved in Virulence

**DOI:** 10.1371/journal.ppat.1009604

**Published:** 2021-05-28

**Authors:** Julia Phenn, Jan Pané-Farré, Nikolai Meukow, Annelie Klein, Anne Troitzsch, Patrick Tan, Stephan Fuchs, Gabriel E. Wagner, Sabine Lichtenegger, Ivo Steinmetz, Christian Kohler

**Affiliations:** 1 Friedrich Loeffler Institute of Medical Microbiology, University Medicine Greifswald, Greifswald, Germany; 2 SYNMIKRO Research Center and Department of Chemistry, Philipps-University Marburg, Marburg, Germany; 3 Department for Microbial Physiology and Molecular Biology, University Greifswald, Greifswald, Germany; 4 Genome Institute of Singapore, Singapore, Republic of Singapore; 5 Duke-NUS Medical School Singapore, Singapore, Republic of Singapore; 6 Cancer Science Institute of Singapore, National University of Singapore, Singapore, Republic of Singapore; 7 FG13 Nosocomial Pathogens and Antibiotic Resistances, Robert Koch Institute, Wernigerode, Germany; 8 Institute of Hygiene, Microbiology and Environmental Medicine, Medical University Graz, Graz, Austria; Children’s Hospital Boston, UNITED STATES

## Abstract

*Burkholderia pseudomallei*, the etiological agent of melioidosis in humans and animals, often occupies environmental niches and infection sites characterized by limited concentrations of oxygen. Versatile genomic features enable this pathogen to maintain its physiology and virulence under hypoxia, but the crucial regulatory networks employed to switch from oxygen dependent respiration to alternative terminal electron acceptors (TEA) like nitrate, remains poorly understood. Here, we combined a Tn*5* transposon mutagenesis screen and an anaerobic growth screen to identify a two-component signal transduction system with homology to RegAB. We show that RegAB is not only essential for anaerobic growth, but also for full virulence in cell lines and a mouse infection model. Further investigations of the RegAB regulon, using a global transcriptomic approach, identified 20 additional regulators under transcriptional control of RegAB, indicating a superordinate role of RegAB in the *B*. *pseudomallei* anaerobiosis regulatory network. Of the 20 identified regulators, NarX/L and a FNR homolog were selected for further analyses and a role in adaptation to anaerobic conditions was demonstrated. Growth experiments identified nitrate and intermediates of the denitrification process as the likely signal activateing RegAB, NarX/L, and probably of the downstream regulators Dnr or NsrR homologs. While deletions of individual genes involved in the denitrification process demonstrated their important role in anaerobic fitness, they showed no effect on virulence. This further highlights the central role of RegAB as the master regulator of anaerobic metabolism in *B*. *pseudomallei* and that the complete RegAB-mediated response is required to achieve full virulence. In summary, our analysis of the RegAB-dependent modulon and its interconnected regulons revealed a key role for RegAB of *B*. *pseudomallei* in the coordination of the response to hypoxic conditions and virulence, in the environment and the host.

## Author summary

Oxygen often becomes a highly limiting factor within tissues, in particular during inflammation and abscesses formation, leading to hypoxic or even strictly anaerobic conditions. Consequently, *B*. *pseudomallei*, a highly neglected and difficult to treat human pathogen has to adjust its metabolism accordingly in order to survive in oxygen-restricted environments. Here, we identify key regulators of anaerobic *B*. *pseudomallei* metabolism and demonstrate the fundamental role of the RegAB redox sensing two-component signal transduction system as the master regulator of anaerobic physiology and its extensive effect on virulence. Further investigations of two RegAB-dependend redox regulators, NarL and FNR, allowed us to provide a first comprehensive insight into the complex regulatory network of anaerobic gene expression in *B*. *pseudomallei*. Integrating these results, we propose a regulatory model of gene expression under oxygen-limiting conditions in *B*. *pseudomallei*. The high level of conservation of the RegAB two-component system in the proteobacteria indicates that our findings are relevant for a broad range of bacteria including may important human and animal pathogens.

## Introduction

During respiration, bacteria use the available electron acceptors in a strict redox potential dependent hierarchy, with oxygen being preferred, followed by nitrate, fumarate and other electron acceptors to guarantee an optimal energy production and bacterial growth. Regulation of the sequential use of available electron acceptors is often realized by a common family of CRP/FNR proteins (cAMP receptor protein/ fumarate and nitrate reduction). This protein family consists of mostly auto-regulated, one-component transcriptional regulators with a conserved C-terminal helix-turn-helix DNA binding domain and a variable N-terminus, which senses a broad spectrum of metabolic co-factors like cAMP, oxygen, nitrogen, or heme [1,2]. CRP regulators are crucial for the control of catabolite repression [3], whereas FNRs are usually involved in oxygen-regulated gene expression [1,4]. In the presence of oxygen, FNR proteins are inactive monomers. When oxygen becomes limited, FNR monomers interact via four N-terminal cysteine residues with [4Fe±4S] clusters to form homodimers. Upon dimerization, FNR binds conserved sequences at specific target promoters to initiate transcriptional activation or repression [2,5–10]. In contrast to FNR, the two-component signal transduction system (TCSTS) ArcAB of *Escherichia coli* senses the redox state of cells by monitoring the ubiquinone pool of the respiration chain [11]. During anaerobiosis, accumulated ubiquinol reduces disulfide bonds located in the Per-Arnt-Sim (PAS) domain of the ArcB sensor kinase thereby stimulating the ArcB kinase activity, which phosphorylates the response regulator ArcA. Phosphorylated ArcA then inhibits the expression of genes for the aerobic metabolism and transcriptionally activates genes required during anaerobic respiration [12–14]. In addition to ArcAB and the FNR regulatory systems, further two-component systems including NarX/L and NarQ/P are involved in the transcriptional control of genes responsible for nitrate/nitrite usage as alternative electron acceptor. Many genes regulated by FNR are also influenced by the ArcAB and/or the Nar TCSTS, leading to a sophisticated network controlling the flow of electrons during energy production. Among photosynthetic and nonphotosynthetic α- and γ-proteobacteria, the TCSTS RegAB constitute a further highly conserved global regulatory system for the redox status dependent control of a variety of energy-generating and energy-utilizing processes such as photosynthesis, CO_2_ fixation, N_2_ assimilation, hydrogen utilization, denitrification, respiratory electron transport and aerotaxis [15]. The ubiquinone pool and additional multiple thiol modifications were identified as redox signal detected by the membrane-bound sensor kinase RegB, similar to what was reported for the ArcB sensor kinase [11,16,17]. Aside of their function in metabolic adaptation to low oxygen tension, redox-sensing regulators are often also important fitness factors of many important pathogens [8,18–22].

*Burkholderia pseudomallei* is a highly pathogenic bacterium that causes melioidosis, a severe disease with a high fatality rate [23–25]. Melioidosis is endemic in the tropics, including regions in East and South Asia, the Middle East, Africa, Latin America, the Caribbean and the Pacific [26]. The bacterium *B*. *pseudomallei* is commonly found in soil and surface water, but is also able to invade and kill almost all types of eukaryotic cells [27]. In the environment, but also during the infection process, *B*. *pseudomallei* often encounters microenvironments that limit or completely prevent aerobic respiration (*e*.*g*. abscesses during an infection) [28]. It is well established that *B*. *pseudomallei* can survive and grow under anaerobic conditions in the presence of suitable terminal electron acceptors (TEA) [25,28]. However, the transcriptional network that enables *B*. *pseudomallei* to coordinate gene expression during hypoxia is completely unknown. In the present study, we performed a transposon mutagenesis screen and identified a RegAB TCSTS homolog being essential for anaerobic growth of *B*. *pseudomallei*. We created a deletion mutant of the response regulator RegA and show that the sensor kinase RegB and the response regulator RegA constitute the master redox-responding global TCSTS in the Betaproteobacterium *B*. *pseudomallei*. We further show, that RegAB is essential for growth under hypoxic conditions with nitrate as TEA and indispensable for full *in vitro* and *in vivo* virulence. Microarray analysis of a *regA* mutant revealed that the *B*. *pseudomallei* RegA regulon is connected to further globally acting redox regulators like the NarX/L TCSTS and a FNR (BPSS0031) homolog. To confirm this observation, mutants in *narL*, *fnr* and other members of the RegAB regulon were created and used in microarray experiments to propose a model for the regulation of anaerobic gene expression in *B*. *pseudomallei*. In addition, we demonstrate that RegAB, but not NarL and FNR homolog BPSS0031 are required for full virulence in cell infection assays and a mouse pathogenicity model. In summary, we described for the first time the core set of transcriptional regulators involved in the adaptation to oxygen-limiting conditions of *B*. *pseudomallei* and confirm their respective role in virulence of this important pathogen.

## Results

### Tn*5* transposon disruption identifies a putative RegB sensor kinase essential for anaerobic growth in *B*. *pseudomallei*

**Fig 1:**
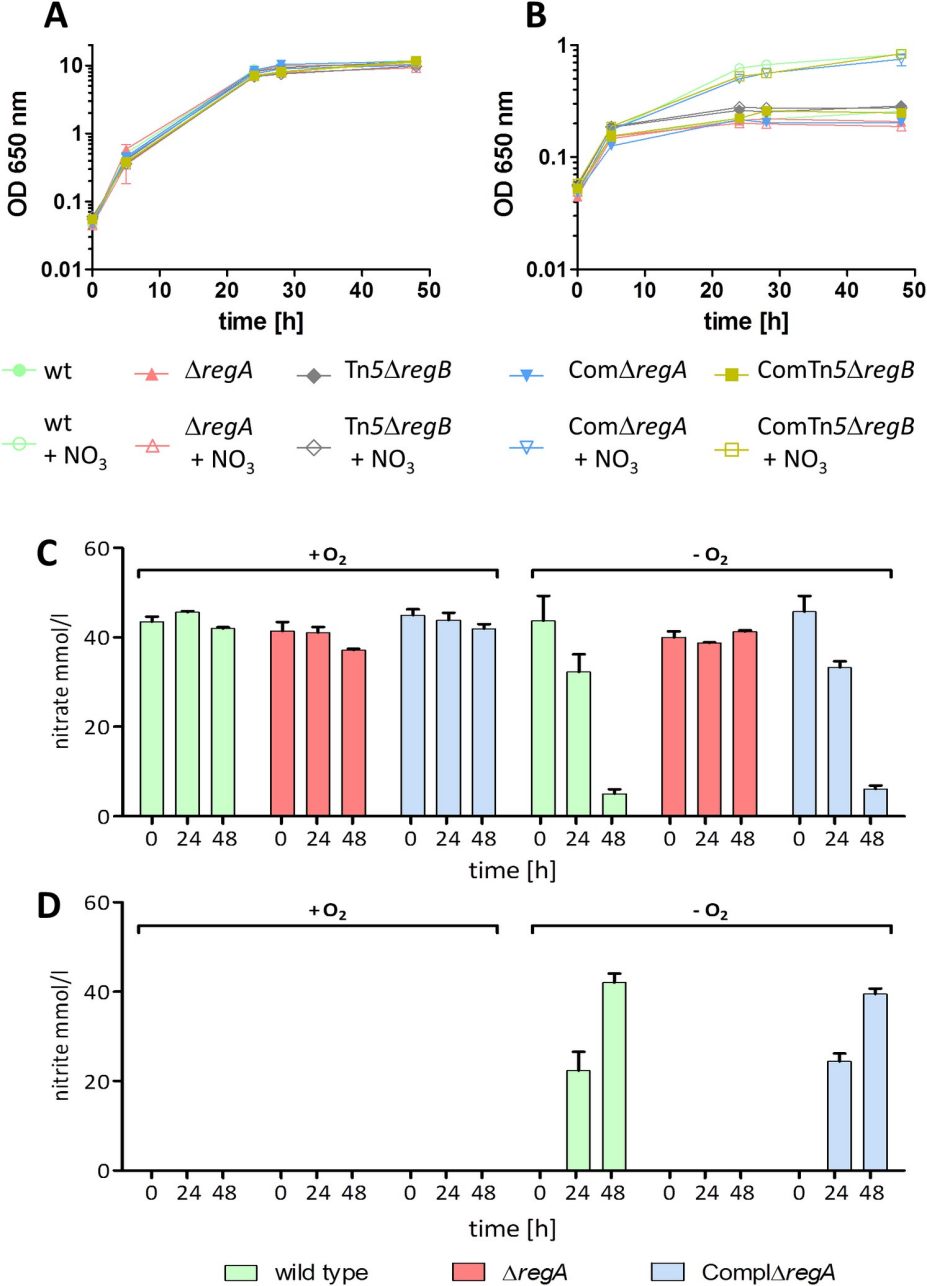
Growth behavior of *B*. *pseudomallei* wild type, Δ*regA* and Tn*5*Δ*regB* mutant, complemented Δ*regA* mutant ComplΔ*regA* and complemented Δ*regB* mutant ComTn5Δ*regB* and the determination of extracellular nitrate and nitrite of *B*. *pseudomallei* wild type strain, Δ*regA* mutant and complemented mutant strain ComplΔ*regA*. A and B: Cells were cultivated in LB medium under aerobic (A) and anaerobic (B) conditions with/without 50 mM nitrate at 37°C and 140 rpm over a time period of 48 hours. Shown are mean values of three independent experiments. Error bars indicate standard error of the mean (SEM). C and D: Cells were aerobically and anaerobically cultivated in LB medium supplemented with 50 mM nitrate. Supernatants were harvested and concentrations of nitrate (C) and nitrite (D) were determined as described in Material and Methods. Shown are mean values of three independent experiments. Error bars indicate the standard error of the mean (SEM).

We screened 2344 *B*. *pseudomallei* transposon mutants for defects in anaerobic growth with nitrate as terminal electron acceptor (TEA) and identified 16 Tn*5* mutants showing growth defects under anaerobic conditions (S3 Table) [29,30]. The majority of mutants with an impaired anaerobic growth with nitrate as TEA had Tn*5* insertions in genes responsible for biosynthesis of cofactors like molybdenum and coproporphyrinogen, electron transport, protein modification, enzymes of unknown specificities or signaling (S3 Table). We focused on a predicted TCSTS, BPSL0201-0202, which we demonstrated to be essential for growth under hypoxic conditions (Figs 1A and S1A and S1B). *In silico* analyses revealed homologies of BPSL0201 to RegB sensor kinases (SK) of different bacterial species (S2A Fig). BPSL0201 contains highly conserved domains specific for RegB SK, six N-terminal located transmembrane domains, the fully conserved heptapeptide sequence GGXXNPF important for ubiquinone binding, the H-box embedded in the dimerization domain and the redox box with a highly conserved cysteine [15,17,31]. The closely related bacteria *Burkholderia thailandensis* codes for a homolog of *B*. *pseudomallei* RegB with an amino acid identity of 99.1% being essential for growth under denitrifying conditions [32]. In *Burkholderia cenocepacia*, a RegB homolog shows an identity of 92.2%, but the physiological role for *B*. *cenocepacia* is still not clear [33]. Interestingly, the adjacent response regulator (RR) BPSL0202 has a higher identity (43.8% to 52.3%) to other RegA RR than the putative SK BPSL0201 to RegB homologs (26.2% to 32.9%) (S2B Fig). In species such as *Rhodobacter capsulatus*, *Caulobacter crescentus or Mesorhizobium loti* the *regB-regA* genes are duplicated and classified as group1 and group 2 RegAB TCSTS [15]. Our sequence analysis identified the RegAB TCSTS of *B*. *pseudomallei* as a member of group 2 (S3A and S3B Fig). While in some species the *regA* and *regB* genes are linked in a single putative *regB-regA* operon (*B*. *pseudomallei*, *C*. *crescentus* or *R*. *capsulatus*) (S1B Fig), both genes can also be part of distinct transcription units [15].

The complemented transposon mutant strains showed the same growth characteristics as the wild type strain (Figs 1A and S1A), indicating that a disruption of *regB* caused by the transposon resulted in the described phenotype. In summary, we identified BPSL0201-BPSL0202 as a RegAB TCSTS homolog in *B*. *pseudomallei* and therefore renamed the genes BPSL0202 to *regA* and BPSL0201 to *regB*.

### Δ*regA* mutant is unable to grow with nitrate under anaerobic conditions

To exclude any polar effects do to Tn*5* insertion in the *regB* gene and to reduce the identification of non-RegAB specific targets do to potential cross talk between the RegB SK and other noncognate RR, we focusing on the RR RegA and constructed a markerless deletion mutant of the corresponding RR RegA (BPSL0202). First we verified results obtained from experiments using the transposon Δ*regB* mutant (Figs 1A and S1A) and performed growth experiments with the Δ*regA* mutant under aerobic and anaerobic conditions. Under normoxia, we observed no differences in growth between wild type, Δ*regA* deletion mutant, Δ*regB* transposon or the complemented Δ*regA* and Δ*regB* mutant strains in the presence or absence of nitrate (Fig 1A). All strains reached a final optical density (OD_650nm_) of about 10. Contrary, in the absence of oxygen and nitrate, none of the strains grew higher than to an optical density (OD_650nm_) of ~ 0.2–0.3. However, in the absence of oxygen but presence of nitrate, only the Δ*regA* and Δ*regB* mutants showed significantly reduced growth (OD_650nm_ of about 0.200–0.250), whereas the wild type and the complemented strains grew to significantly higher optical densities (OD_650nm_ of about 1), showing that both, RegA and RegB are essential to use nitrate as a terminal electron acceptor under anaerobic conditions (Fig 1B). To further confirm this, we monitored the nitrate consumption and nitrite secretion of the wild type, the Δ*regA* mutant and the complemented Δ*regA* strain (Fig 1C and 1D). During aerobic growth no nitrate was consumed and no nitrite was produced by neither of the three strains. Under hypoxia with nitrate as the TEA however, only the wild type and complemented strain consumed nitrate and simultaneously secreted nitrite in the supernatant (Fig 1C and 1D). We further determined the ability to grow with nitrite instead of nitrate as electron acceptor. As observed for nitrate, the Δ*regA* mutant did not grow anaerobically with nitrite as TEA and consequently did not show consumption of nitrite (S10 Fig). Thus, we assume that the arrested growth of the Δ*regA* and Δ*regB* mutant under oxygen deficiency is caused by the inability to metabolize nitrate and/or nitrite.

### Δ*regA* mutant is severely attenuated in a murine infection model

**Fig 2:**
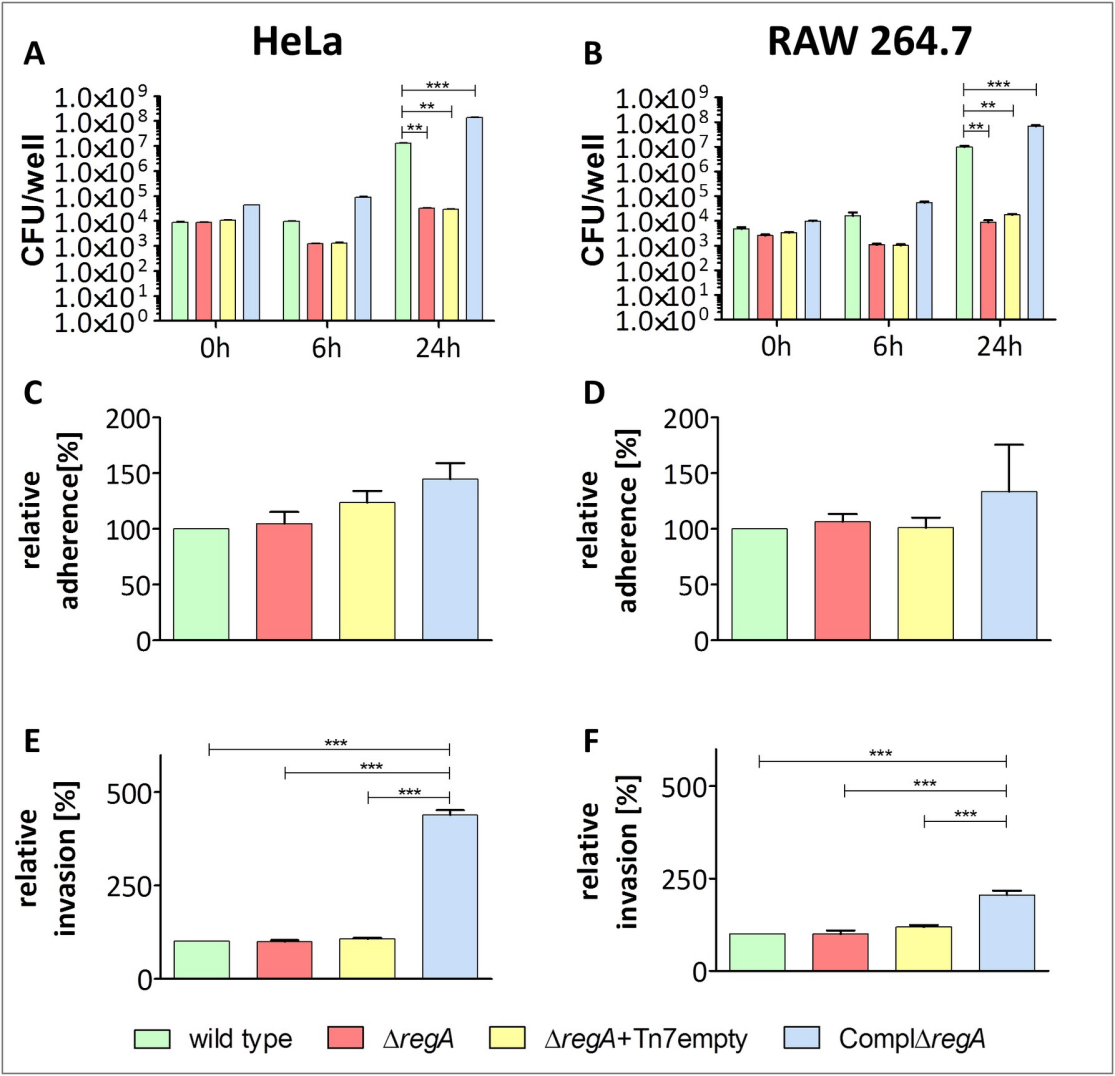
Replication (A, B), relative adherence (C, D) and relative invasion (E, F) of *B*. *pseudomallei* strains in HeLa- T cells and RAW264.7 macrophages. Wild type strain, Δ*regA* mutant, Δ*regA*+Tn7empty (mutant with an empty pUC18T-mini-Tn7-Km-FRT plasmid) and the complemented mutant ComplΔ*regA* were cultivated aerobically on blood agar for 16 hours at 37°C. Then, cells were diluted in PBS and used at MOI 10 with HeLa- T cells (A, C, E) and MOI 5 with RAW264.7 macrophages (B, D, F) for infections. A and B (intracellular replication): At indicated time points (0, 6, 24 hours), the infected Hela- T and RAW264.7 cells were lysed and dilutions were plated on LB agar plates. After 48 h of growth at 37°C colonies of *B*. *pseudomallei* on the plates were counted and CFU/well were calculated. Shown are mean values of three independent experiments. Error bars indicate standard error of the mean (SEM). C, D, E and F (relative adherence and invasion): For determination of adhesion, numbers of adherent bacteria were calculated at 30 minutes post-infection. For determination of invasion, numbers of invasive bacteria were calculated at 30 minutes post-infection plus 30 minutes after antibiotic protection assay. The percentage of adhesion and invasion represented the number of intracellular bacteria relative to their inoculums compared to the wild type. Shown are mean values of three independent experiments. Error bars indicate standard error of the mean (SEM). Statistical analyses were performed by using ONE- way- ANOVA followed by Bonferroni post- hoc test (* *p* < 0.05; ** *p* < 0.01, ***p < 0.001).

**Fig 3:**
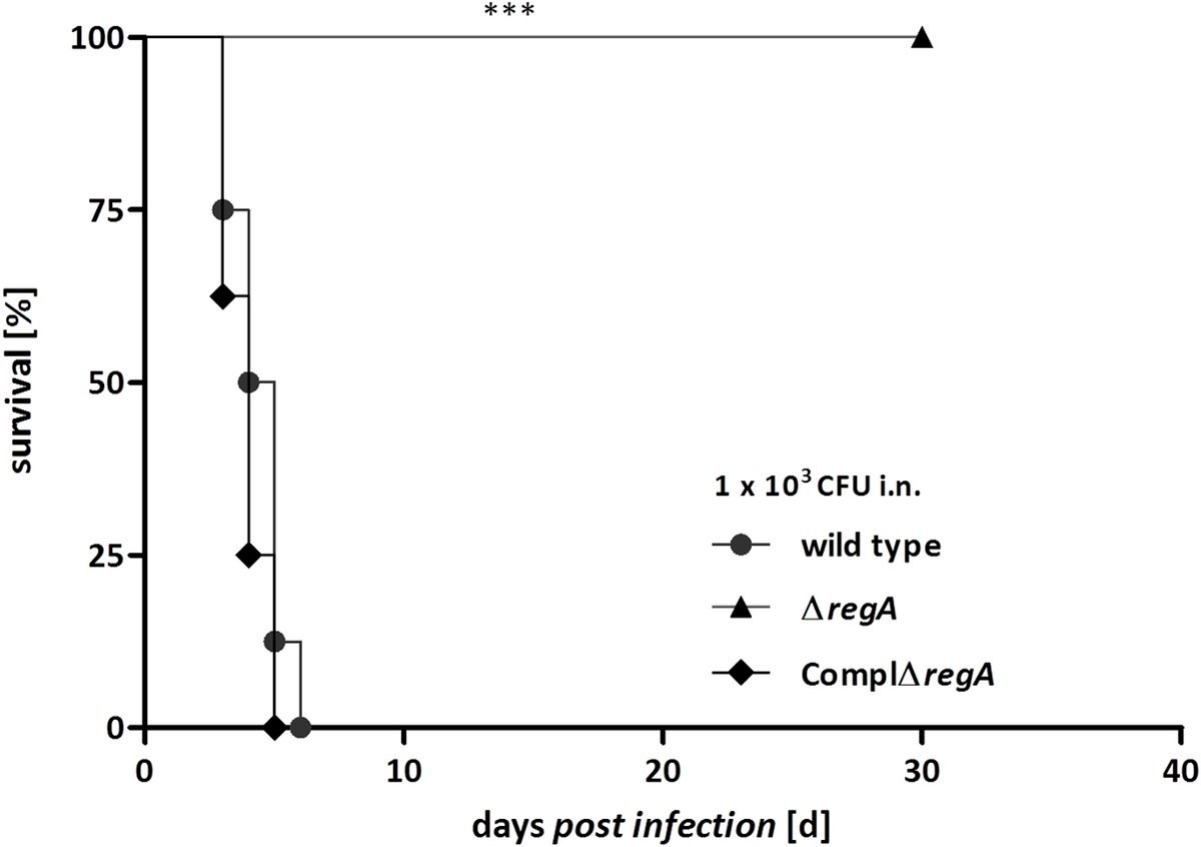
Mortality curves of Balb/ c mices infected with the *B*. *pseudomallei* wild type, Δ*regA* mutant and complemented mutant ComplΔ*regA*. Mice (n = 8) were i.n. infected with 1000 CFU. Pooled data from two independent experiments are shown. Curves were compared by using the log rank Kaplan- Meier test (*** *p* < 0,001).

**Fig 4:**
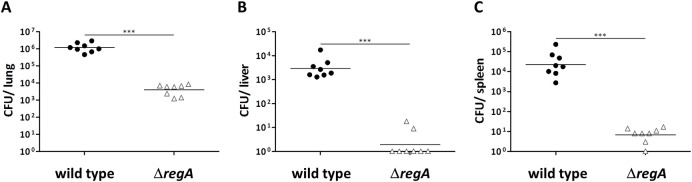
Determination of bacterial loads in lung (A), liver (B), and spleen (C) 48 h after i.n. infection of mice (n = 8) with ∼100 CFU *B*. *pseudomallei* wild type or Δ*regA* mutant. Shown are pooled data of two independent experiments. Significant differences were calculated using Student’s t test with Welch´s correction.(*** *p* < 0,001).

To examine if the reduced fitness under anaerobic conditions has an impact on virulence, we used *in vitro* cell assays to compare intracellular replication, cell adhesion and invasion capacity of the wild type, the Δ*regA* mutant and the complemented strain. To exclude any polar effects caused by the plasmid used for complementation, we included a Δ*regA* mutant strain containing the empty pUC18T-mini-Tn7 vector as a control (Δ*regA*+Tn7empty). The wild type and complemented Δ*regA* strain showed strong intracellular replication in the two tested cell lines (human derived HeLa cells and murine RAW264.7 macrophages). Contrary, the replications of the Δ*regA* mutant and the Δ*regA* vector control were clearly reduced in both, HeLa cells and RAW264.7 macrophages (Fig 2A and 2B). Determinations of the bacterial adherence and invasions to HeLa and RAW 264.7 cells revealed no differences between the wild type and both Δ*regA* mutant strains (Fig 2C, 2D, 2E and 2F). Only the complemented strain showed increased cell adherence and invasion, explaining their slightly higher intracellular cell numbers after 6 and 24 hours compared to the wild type strain. Together, these results clearly showed that the decreased intracellular replication of Δ*regA* mutant is not caused by a changed ability to adhere or invade the two tested cell types. Finally, we investigated in vivo virulence of the Δ*regA* deletion mutant, the Δ*regB* transposon mutant and the respective fully complemented mutant strains in mouse models of infection (Figs 3 and S1C). Both Δ*regA* and Δ*regB* mutants showed a completely attenuated lethality and demonstrated the importance of the RegAB TCSTS for *B*. *pseudomallei* pathogenicity. These results were supported by determining the bacterial loads in lung, spleen and liver 48 hours after infection using the wild type and Δ*regA* mutant. We observed high bacterial loads in organs infected by the wild type and strongly reduced bacterial loads for the Δ*regA* mutant (Fig 4). Together, these results showed that a disruption of the RegAB TCSTS led to a strong attenuation in virulence and renders the pathogen unable to cause disease under the conditions tested.

### RegA influences gene expression of further global regulators of anaerobic metabolism

To gain a deeper insight into the physiology and virulence phenotype of the Δ*regA* mutant, we compared the gene expression profiles of the Δ*regA* mutant to that of the parental strain E8 using full genome high-density tiling arrays. Transcriptome analysis of the bacteria cultivated anaerobically either in LB medium or LB medium supplemented with 50 mM nitrate identified a surprisingly large number of putative transcriptional regulators whose transcription was either repressed (16 genes) or induced (4 genes) in the Δ*regA* background, but only if cultivated with nitrate as TEA (S2 Table). In nitrate free medium, we only found 4 regulator genes with a transcriptional repression (S2 Table). Of special interest were three genes encoding for putative CRP/FNR proteins (BPSS0031, BPSS1163 and BPSS1917). While repression of BPSS0031 in the Δ*regA* background was observed independent from nitrate availability, repression of BPSS1163 and BPSS1917 was only observed in the presence of nitrate (S2 Table). At the protein level BPSS0031 has an identity of 99.2% to FNR from *B*. *thailandensis*, 42.5% to FNR from *E*. *coli* and 25.2% to FNR from *Bacillus subtilis*. In *B*. *pseudomallei*, BPSS1917 and BPSS1163 are putative paralogs to BPSS0031, with 55.7% and 56.5% identity to BPSS0031, respectively. Also strongly repressed by a disruption of the *regA* gene was the TCSTS BPSL2313-BPSL2314, which shows similarity to the NarX/L TCSTS from *B*. *thailandensis* (96.1% and 97.0% respectively) and from *E*. *coli* (29.0% and 47.0% respectively). As observed for transcription of the putative *fnr* (BPSS0031), transcriptional repression of the *narL* (BPSL2314) homolog in the *regA* mutant was independent from nitrate supplementation.

**Fig 5:**
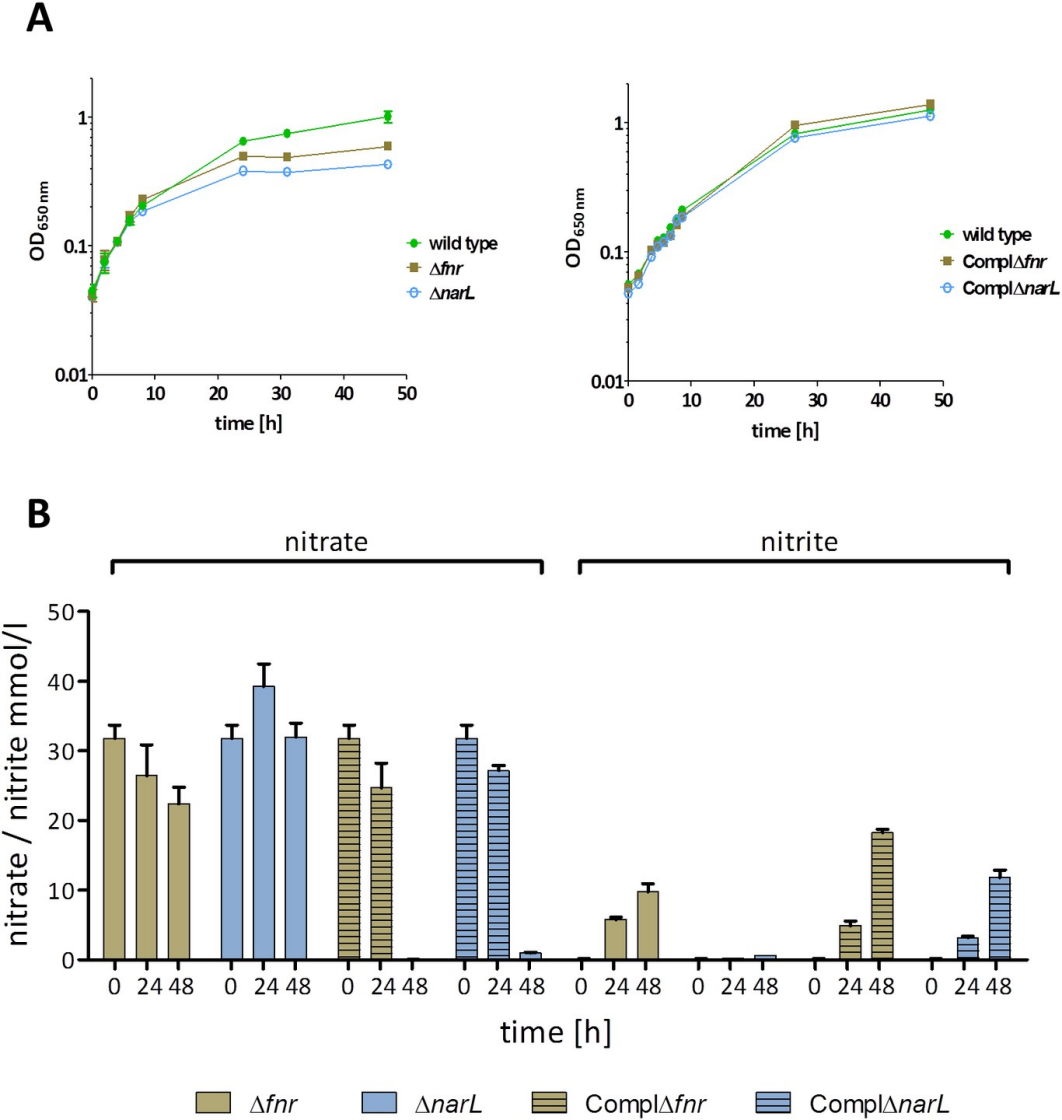
Growth and determination of extracellular nitrate and nitrite of *B*. *pseudomallei* wild type strain, Δ*fnr* and Δ*narL* mutants and their respective complemented mutant strains. A: For growth curves, cells were anaerobically cultivated in LB medium supplemented with 50 mM nitrate. B: For the determination of nitrate and nitrite, the supernatants were harvested and concentrations of nitrate and nitrite were measured as described in Material and Methods. Shown are mean values of three independent experiments. Error bars indicate standard error of the mean (SEM).

Members of the FNR and Nar protein family are often essential regulators for a successful adaptation to hypoxic conditions. Therefore, to investigate if these previously uncharacterized regulators contribute to anaerobic gene expression in *B*. *pseudomallei* we deleted the corresponding genes and characterized growth behavior of the mutants under aerobic and anaerobic conditions. Confirming an important role in anaerobic gene expression, we observed a strong growth arrest of the Δ*narL* mutant and highly impaired growth of the FNR deficient strain under hypoxia with nitrate as TEA, whereas growth of the complemented mutants was fully restored (Fig 5A). No differences were seen under aerobic conditions, similar as observed for the Δ*regA* mutant (S4 Fig). Monitoring of nitrate consumption revealed a total inability of the Δ*narL* mutant and a delay of the Δ*fnr* mutant in nitrate utilization (Fig 5B).

### Transcriptome alterations in *B*. *pseudomallei* Δ*regA*, Δ*narL* and Δ*fnr*

**Fig 6:**
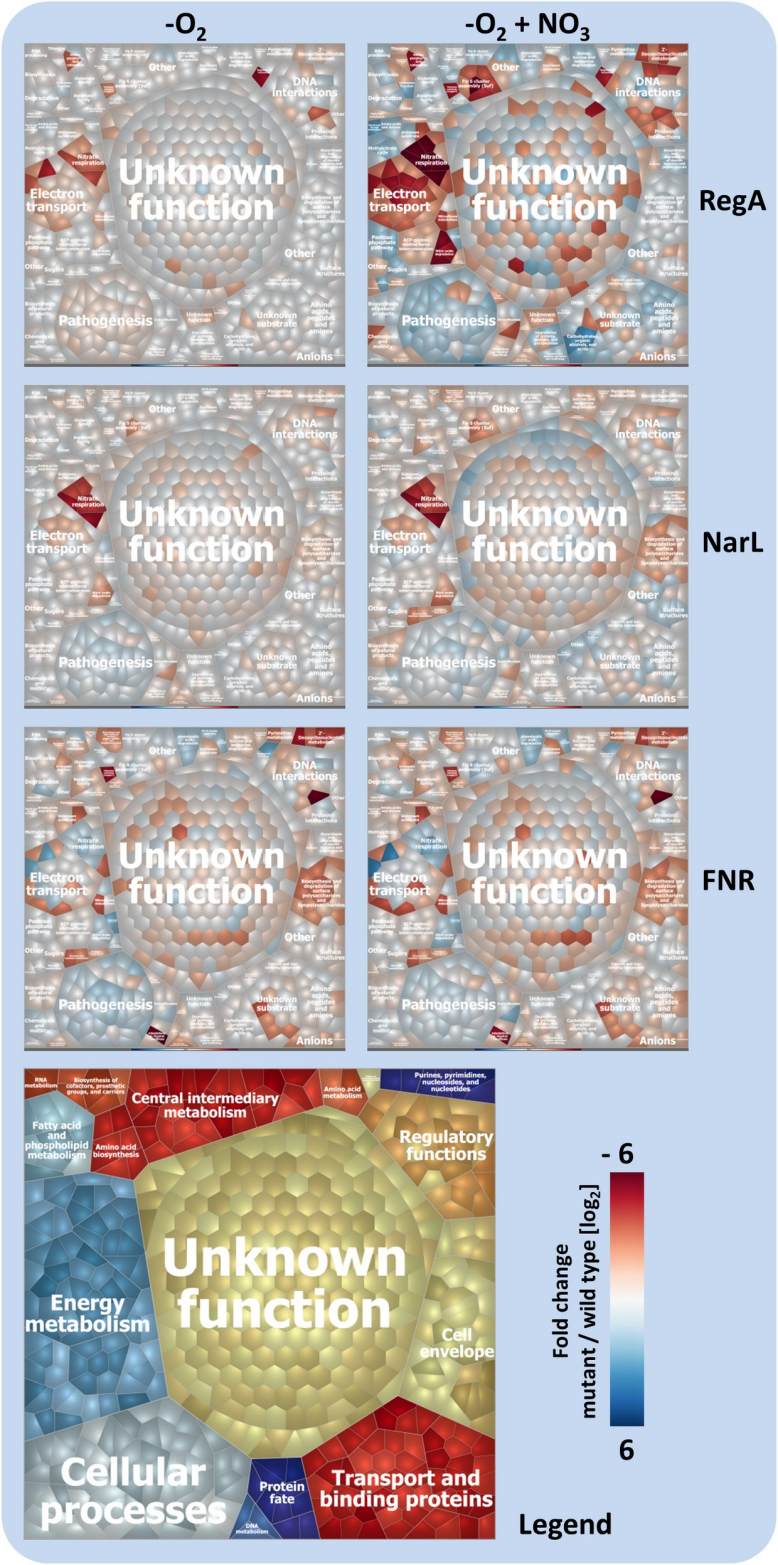
Voronoi treemap visualize RegA, NarL and FNR dependent gene expressions under hypoxia in absence (left) or presence of nitrate (right). Shown are the microarray based fold changes of the respective mutant strains versus the wild type. Pathways given in orange-brownish background are down-regulated, pathways in blue colours are up-regulated in mutant strains. Darker colors represent greater differences in gene expression. Treemaps were built using the Paver software (DECODON GmbH, Germany) on the basis of functional annotation validated on the basis of sequence similarity and domain analysis according KEGG (https://www.genome.jp/kegg/).

To further characterize the regulatory network controlling anaerobic gene expression in *B*. *pseudomallei* we performed global transcriptome analyzes of the Δ*narL* and Δ*fnr* strain under hypoxia with and without nitrate as TEA and compared the results with the transcriptome of the Δ*regA* mutant (Fig 6 and S2 Table). Results of the DNA microarray experiments were confirmed by qRT PCR of 25 selected genes and are shown in S9 Fig.

**Fig 7:**
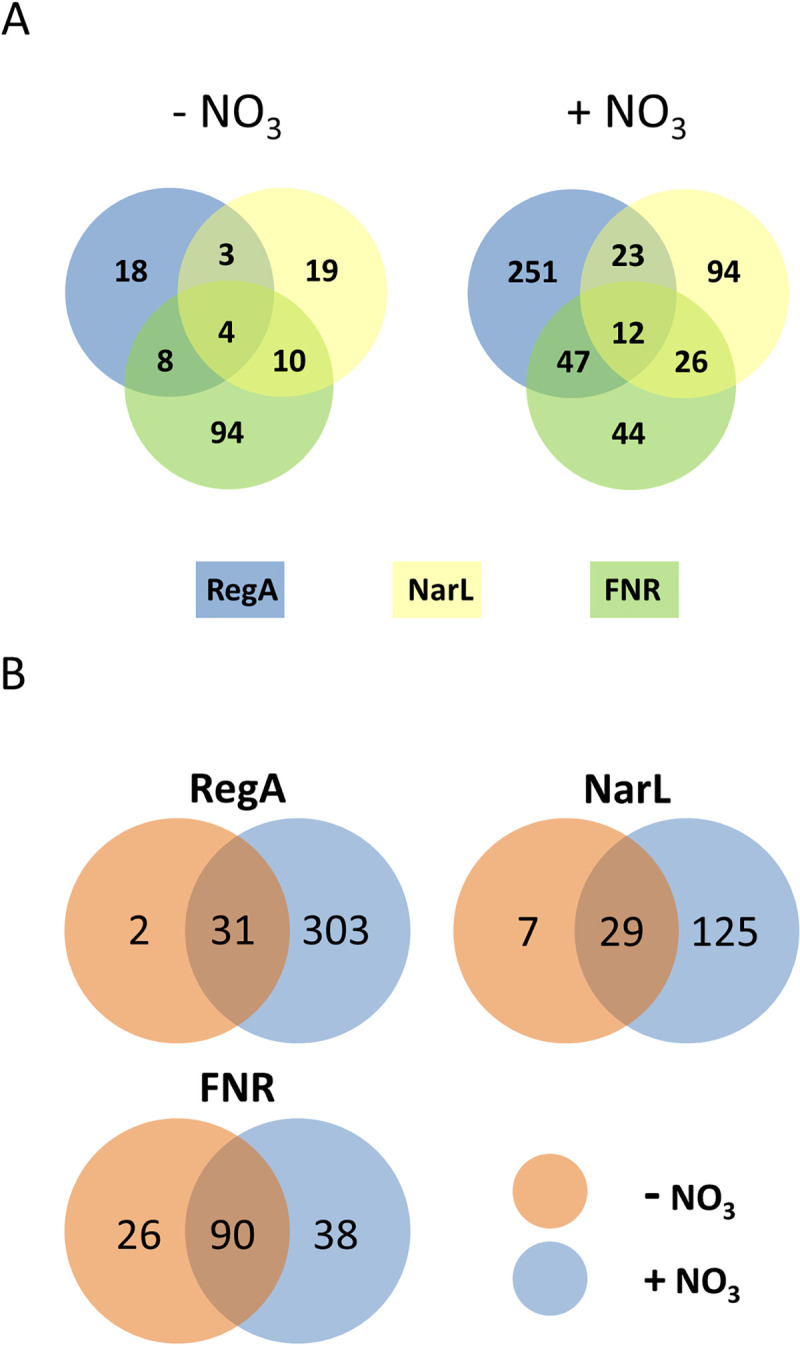
Influence of nitrate (NO_3_^-^) on the number of RegA, NarL and FNR dependent gene expressions. A: VENN Diagramm presenting the number of genes affected by the regulatory interactions between RegA, FNR and NarL obtained from global gene expression experiments. B. VENN diagrams show the total numbers of either RegA, NarL or FNR regulated genes in absence (orange) and presence (blue) of NO_3_^-^. The overlaps represent the genes influenced under the respective regulators RegA, NarL and/or FNR and nitrate absence or presence (A) or only under both growth conditions (B).–NO_3_^-^: growth without nitrate as TEA, + NO_3_^-^: growth with nitrate as TEA.

In total, 517 genes exhibited an at least 2-fold differential expression in at least one of the three mutants as compared to the wild type, when cultured under anaerobic conditions in the absence or presence of nitrate (S2 Table). In nitrate free medium, only 157 genes showed altered transcription, whereas in nitrate containing LB medium 497 genes showed a changed expression (Fig 7A and S2 Table). The nitrate-dependent changes in transcription were mainly dependent on RegA and NarL, while mutation of the two regulators had only a minor effect on anaerobic gene expression in the absence of nitrate (Figs 6 and 7B). As shown in S2 Table, in the Δ*regA* mutant only 3 genes were induced and 30 genes were repressed in nitrate free growth conditions, whereas 143 genes showed increased and 191 genes a decreased expression when cultivated with nitrate. In the Δ*narL* mutant, 63 genes were induced and 91 repressed in LB medium with nitrate, but only 3 genes showed increased and 33 genes decreased expressions in this mutant when nitrate was absent (S2 Table). In contrast, FNR dependent gene expression was mainly dependent on oxygen availability, since the majority of genes with changed transcriptions in the *fnr* mutant were also observed in the absence of nitrate (Figs 6 and 7 and S2 Table).

To further elucidate the regulatory interactions between RegA, FNR and NarL in controlling anaerobic physiology, we focused our analysis on gene expression profiles under hypoxia and with nitrate as TEA. Under this growth condition, the majority of the regulated genes (326 genes = 65.5%) encode proteins with known or predicted function, including processes like denitrification, general anaerobic metabolism pathways, different dehydrogenases and electron transport systems, nutrient transporters or stress resistance. The remaining 172 (34.5%) genes have unknown functions (S2 Table). We classified the regulated genes according to the level of influence caused by mutations in *regA*, *narL* and *fnr* (S2 Table), with group 1 including genes affected by all three regulators, groups 2, 3 and 4 including genes affected by two regulators (RegA-NarL, RegA-FNR or NarL-FNR) and groups 5, 6 and 7 including genes showing changed expression in only one of the tested backgrounds (S2 Table).

The transcription of genes involved in denitrification, NO stress response and iron-sulfur-cluster assembly are highly affected by RegA, NarL and FNR. Genes of this group are highly dependent on RegA and NarL as activators, whereas FNR acts as a repressor (S2 Table). During cultivation without nitrate, only four genes (BPSL2307-2309 and BPSL2312) were found to be dependent on all three regulators. The nitrate-dependency was particularly obvious for genes affected by the *regA* mutation, while transcription of many other genes was still observed in the absence of nitrate in the *fnr* and *narL* mutant.

Both, RegA and NarL but not FNR are necessary for the transcriptional activation of genes coding electron transporter, the SUF apparatus, nitrite reduction forming nitric oxide (NO) (BPSS1487) and the NO sensing (BPSL2376). Since nitrite is toxic for cells, a fast conversion to NO by BPSS1487 and later to nitrous oxide (N_2_O) by BPSL2351 and/or BPSL2368 is necessary. RegA and FNR but not NarL strongly influenced the gene expressions of genes coding mainly electron transport systems (*cyoABCD* (BPSL2378-2381 and BPSS1897-1894), cytochrome *bd* ubiquinol oxidase complex *cydAB* (BPSS0235-0234)), the nitrous-oxide reduction via NosZ (BPSL1607)) and the anaerobic purine/pyrimidine synthesis (anaerobic ribonucleoside triphosphate reductase complex (BPSL2356-2358). While all of the corresponding genes are under a strong positive control of RegA and FNR, only the cytochrome *bd* ubiquinol oxidase complex *cydAB* (BPSS0235-0234) was positively regulated by RegA but repressed by FNR. NarL and FNR but not RegA positively affected the transcription of genes being involved in the metabolism of fructose and mannose, amino and nucleotide sugars and exopolysaccharide (EPS).

RegA alone influenced the gene expression of 252 genes with a high functional diversity (S2 Fig). As mentioned above, the high number of genes with changed expression in the Δ*regA* mutant could be a consequence of 16 genes encoding further regulators or regulatory systems (including global regulators like NarXL and FNR) changed in this background. All other up- and downregulated genes are involved in energy metabolism, electron transport, transport of carbohydrates and amino acids, central intermediary metabolisms, ATP synthesis and pathogenesis. Of note, a second cytochrome *bd* ubiquinol oxidase complex CydAB (BPSL0501-0502) was repressed independently of nitrate in the Δ*regA* mutant. Also repressed were two paralogs of the coproporphyrinogen III oxidase *hemN* (BPSL2646 and BPSL2366), enzymes that belongs to the radical SAM superfamily and contains a 4Fe-4S cluster coordinated by a SAM molecule [34–36]. Other genes were found to be repressed by RegA like genes involved in the transport of carbohydrates, amino acids and ions (BPSL1277-1278, BPSL2535, BPSL2608-2611, BPSL2615-2617) or acting in central energy metabolism pathways (methylcitrate cycle–BPSS0206-0208, pentosephosphate cycle–BPSL2612-2614 and BPSL2931-2932). Under nitrate free conditions, gene expressions of only three regulator coding genes (*narXL*, *fnr* and BPSS0730) and further 14 genes were influenced by Δ*regA* mutation.

NarL alone affected the transcription of four additional regulators which may explain the higher number of genes influenced by NarL with nitrate as TEA, since only one additional regulator (BPSS1214) is under positive control of NarL in nitrate absence. NarL further influenced the transcription of genes belonging to different functional groups like LPS and peptidogylcan metabolism, transport but also of an unknown secondary metabolism cluster (BPSS1192-1197).

FNR alone controls many genes of the anaerobic metabolism independent from nitrate presence, which indicates that the activity of FNR is probably only dependent on oxygen availability. However, in nitrate presence, many of FNR-regulated genes were additionally influenced by *regA* and/or *narL* deletions.

### RegA also positively influences transcription of genes under aerobic conditions

As mentioned before, *in vivo* experiments suggest an essential role of the RegAB TCSTS in virulence and therefore imply an important role even under normoxic to microaerophilic conditions. We therefore included transcriptome studies of the Δ*regA* mutant cultivated in LB media with normal aeration. Under this circumstance, RegA positively influenced gene expression of 13 genes of different functional categories, including *narX*, *fnr*, *cydAB* (BPSS0235-0234), *cyoAB* (BPSS1897-1896), the *cyoA* homolog BPSL2378, *hemN* (BPSL2646) and few other genes (Table 1), but their induction rates were lower than observed as under anaerobic conditions. Together, this clearly shows the global impact of the RegAB TCSTS in *B*. *pseudomallei*, even in the presence of oxygen.

**Table 1:** Expression analysis of RegA-dependent genes upon normoxic conditions.

Gene locus^*a*^	Gene name^*a*^	Definition^*a*^	Ratio wt vs. Δ*regA* [log_2_]^*b*^	Mainrole^*c*^	Subrole^*c*^
BPSL0503	*-*	hypothetical protein	1,16	Energy metabolism	Electron transport
BPSL2313	*narX*	putative nitrate/nitrite sensor protein	1,50	Regulatory functions	Protein interactions
BPSL2378	*cyoA*	ubiquinol oxidase polypeptide II precursor	1,76	Energy metabolism	Electron transport
BPSL2412	*-*	SCO1/SenC family protein	1,39	Protein fate	
BPSL2646	*hemN*	coproporphyrinogen III oxidase	2,56	Biosynthesis of cofactors, prosthetic groups, and carriers	Heme, porphyrin, and cobalamin
BPSL3237	*-*	hypothetical protein	1,54	Unknown function	
BPSS0031	*fnr*	anaerobic growth regulatory protein	2,41	Regulatory functions	DNA interactions
BPSS0234	*-*	cytochrome bd ubiquinol oxidase subunit II	3,32	Energy metabolism	Electron transport
BPSS0235	*-*	cytochrome bd ubiquinol oxidase subunit I	4,77	Energy metabolism	Electron transport
BPSS0683	*-*	hypothetical protein	1,43	Unknown function	
BPSS1896	*cyoB*	cytochrome o ubiquinol oxidase subunit I	1,35	Energy metabolism	Electron transport
BPSS1897	*cyoA*	cytochrome o ubiquinol oxidase subunit II	2,44	Energy metabolism	Electron transport
BPSS2003	*-*	conserved periplasmic protein	1,32	Unknown function	

^*a*^—Gene locus, Gene name and Definition were obtained from KEGG

^*b*^—wt = wild type strain E8, Δ*regA* = Δ*regA* mutant

^*c*^—KEGG mainrole and subrole

### Deletions of individual genes involved in anaerobic adaptation revealed their importance for anaerobic fitness but not in virulence

To probe the role of individual stress protective and metabolic genes highly expressed under anaerobic conditions in *B*. *pseudomallei*, single gene deletions were created and analyzed for their impact on growth, nitrate consumption, nitrite secretion, intracellular survival in RAW264.7 macrophages and virulence in BALB/c mice.

First, we disrupted three genes involved in the denitrification process: the respiratory nitrate reductase subunit alpha *narG*, the nitric oxide reductase subunit BPSL2351 (NOR) and the nitrous-oxide reductase *nosZ*. We also deleted *narG2* (BPSS1159), from a second uncharacterized, paralogous respiratory nitrate reductase complex (BPSS1159-BPSS1154) located on chromosome II, showing weak repression in the Δ*regA* mutant. Further we deleted an NnrS homolog BPSL2355, a protein important for resistance to nitrosative stress under anaerobic conditions as described for *Vibrio cholerae* [37, 38]. Finally, we deleted genes/systems are involved in electron transport processes: the oxygen-independent coproporphyrinogen III oxidase *hemN* (BPSL2646) and two highly identical cytochrome *bd* ubiquinol oxidase systems (BPSL0234-0235 and BPSL0502-0501). All mutants were complemented to exclude polar effects.

For all mutant strains, no growth differences were observed under aerobic conditions, independently of nitrate supplementation. Under hypoxia in nitrate free medium the mutant strains did also reveal no differences in growth, while upon nitrate supplementation a strong growth arrest was observed for the Δ*narG* and ΔBPSL2351 mutants (S5 Fig). These results were in concordance with the inability of both mutants to use nitrate as TEA and to secrete nitrite into the LB medium (S6 Fig). All remaining mutants grew comparable to the wild type strain and we determined similar nitrate and nitrite concentrations in the medium. Interestingly, we were unable to complement the ΔBPSL2351 mutant strain, even if the upstream region of BPSL2351 in the complementation strain was increased from 233 to 471 base pairs. The reason for this discrepancy is currently under investigation.

Next we determined the intracellular replication of all these mutant strains and also included the Δ*narL* and Δ*fnr* mutants in this experiment. Almost all strains showed a tendency towards lower intracellular replication in a RAW 264.7 macrophages infection assay under normoxic and hypoxic conditions, however, none of these reductions were significant (S7 Fig). The results suggested that the mutants retained their virulence under the tested *in vitro* conditions. This was confirmed *in vivo*, as we observed no differences in the survival rates between the wild type and the mutant strains in infected BALB/c mice (S8 Fig).

## Discussion

### RegB/RegA of *B*. *pseudomallei* is a Highly Conserved Global Redox-Response TCSTS Regulatory System

*B*. *pseudomallei* thrives deep in soil, but can also infect many different types of eukaryotic cells, habitats often characterized by reduced oxygen concentrations [39–43]. During growth at low oxygen levels a comprehensive reorganization of the *B*. *pseudomallei* transcriptome can be observed but the underlying regulation remains poorly understood [28]. We identified a homolog of the RegAB TCSTS (BPSL201-BPLS0202) and demonstrated its crucial role for *B*. *pseudomallei* during growth in anaerobic conditions. Although the number and diversity of RegAB regulated genes is enormous in different species, the signal sensing mechanisms by RegB is usually conserved [15, 31, 44]. RegB kinase activity is controlled by the ratio of oxidized to reduced ubiquinone molecules, which is sensed by a highly conserved quinone binding motif, GGXXNPF [31]. In addition to the quinone/quinol ratio, modifications of the conserved cytosolic cysteine 265 (Cys-265) controls RegB activity [16]. These features are also present in *B*. *pseudomallei* RegB, suggesting a similar rout of signal transduction [16, 31, 45]. Although RegAB TCSTSs were extensively described for α- and γ-proteobacteria, little is known about their role in ß-proteobacteria [15, 44, 46].

### RegAB modulates the expression of several global regulators orchestrating a complex regulatory network

To characterize in detail the role of the *B*. *pseudomallei* RegAB TCSTS in anaerobic gene expression, we compared the transcriptome of a wild type and a mutant, carrying a deletion of the *regA* RR gene. About 6% of the coding genes showed an altered transcription in the Δ*regA* strain, which is in a same range as reported for RegAB regulons of other species[18, 32, 47].

Importantly, among the RegAB regulated genes we identified many potential regulators including a FNR homolog (BPSS0031) and a NarX/NarL TCSTS homolog. Both systems are well conserved global regulators in the adaptation to hypoxia in many different bacteria, including several *Burkholderia* species [48–53]. Therefore, both genes were deleted to elucidate their individual contribution to anaerobic gene expression within the RegAB modulon. These analyses revealed that the impact on gene regulation of RegAB and NarL was maximal in the presence of nitrate supplement. A large number of genes with a role in nitrate respiration, NO detoxification and the Fe-S cluster assembly were downregulated in both mutants. Consequently, both mutants did not grow in the presence of nitrate during hypoxia.

The nitrate-dependency of RegAB and NarL activity ensures that futile expressions of nitrate respiration in the absence of nitrate as TEA is avoided. The molecular mechanism linking nitrate and RegAB and NarL activity, however, remains to be elucidate in *B*. *pseudomallei*. For *E*. *coli* it was shown that nitrate elicits a strong activation of the NarL associated NarX SK [54,55] and we propose that this activation does also occur in *B*. *pseudomallei* NarX. Metabolism of nitrate leads to production of toxic NO, which in *P*. *aeruginosa* and *E*. *coli* is sensed by Dnr (iron nitrosylations of the Fe-S cluster) and NsrR (reaction with the ferrous heme-iron), respectively [56–62] [63–65]. Homologs of Dnr (BPSL2365) and NsrR (BPSL2376) were also identified as regulated by RegAB in our study. Of note, Dnr (BPSL2365) appears to be essential for growth on nitrate, as we also identified this gene by the transposon mutagenesis (S3 Table). Among the genes downregulated in the *regA* mutant we also identified a second nitrate reductase complex (BPSS1159-BPSS1154) with high homology to the *narZYWV* of *E*. *coli* [52,66,67]. However, while we observed a strong negative effect on growth and nitrate utilization for a *narG* (BPSL2309) mutant, no impact on anaerobic nitrate reduction or growth was observed for the *narG2* (BPSS1159) mutant. This differed from the situation in *B*. *thailandensis*, where it was suggested that NarZYWV can functionally substitute for NarGHJI [68].

An induction of alternative respiration systems is mostly coupled to the expression of specific electron transport systems. Indeed, several predicted electron transport systems were upregulated in a RegA-, NarL- and FNR-dependent manner. The only exception was the FNR-dependent repression of a putative cytochrome *bd* ubiquinol oxidase BPSS0235-0234 with homologies to the AppCB and/or CydAB of *E*.*coli*. Our growth experiments showed that single deletions in the two strongly activated cytochrome *bd* ubiquinol oxidase complexes (BPSS0235-0236 and BPSL0502-0501) did not affect growth under anaerobic conditions, excluding an important role of these two terminal oxidases for anaerobic respiration in *B*. *pseudomallei*. This contrast results reported for other bacteria [69,70,71] and raises the possibility that the targeted terminal oxidases are functionally redundant.

Many respiratory enzymes require heme as a cofactor and we identified two oxygen-independent coproporphyrinogen III oxidase paralogs (HemN; BPSL2366 and BPSL2646) as part of the RegAB modulon [72–74]. Although at the transcriptional level BPSL2646 showed the stronger induction, a growth defect in anaerobic conditions was only observed for BPSL2366 (Tn*5* insertion, see S3 Table). Interestingly, BPSL2366 shows a chromosomal association with a predicted CRP/FNR family regulator of dissimilatory nitrate respiration regulator (BPSL2365). A Tn mutation in BPSL2365, resulted in reduced growth in anaerobic conditions further suggesting an important role for this HemN paralog during nitrate respiration.

Together, our findings show that RegAB is the master regulator controlling the transcription of downstream regulators like NarL, and probably Dnr and NsrR to coordinate the efficient use of nitrate as TEA and to avoid the accumulation of toxic intermediates such as NO during nitrate respiration.

In contrast, to these regulators, we show that FNR dependent gene expression depends mainly on oxygen as shown in other species [7,75–77]. FNR regulated genes differed only moderately between cells grown in LB medium with or without nitrate. This is in accordance with the observed phenotype of the Δ*fnr* mutant, which is still able to growth slightly with nitrate as TEA in anaerobic conditions. The strong repression of 2´-deoxyribonucleotide metabolism genes (BPSL2356-2358) in the Δ*fnr* mutant, which are necessary for DNA replication under anaerobic conditions in *E*. *coli*, likely further supports anaerobic growth behavior of the Δ*fnr* mutant [78,79]. We assume that the Δ*fnr* mutant installed the first steps of nitrate respiration which resulted in the observed slightly higher growth than that of the Δ*regA* and Δ*narL* mutant. After total oxygen consumption it probably cannot compensate the deprivation of deoxyribonucleoside triphosphates and consequently cannot further replicate.

### RegAB, but not the denitrification pathway is essential for virulence in *B*. *pseudomallei*

The metabolic activity of bacterial pathogens and immune cells during abscess formation can lead to completely anaerobic conditions [80–83]. Anaerobic fitness of several microbial pathogens is often required for full virulence and pathogenesis [8,84–87]. It is therefore conceivable that the capacity to adapt to hypoxia during infection is also essential for *B*. *pseudomallei*, since the formation of abscesses in a variety of organs (lungs, liver, and spleen) is a characteristic feature of melioidosis [41]. In this study, we show for the first time that RegAB, the master regulator of anaerobic metabolism in *B*. *pseudomallei*, is absolutely essential for full virulence in human and murine cell lines and in a mice infection model. However, mutations in further global anaerobic regulators *narL* or *fnr*, whose transcription is coregulated by RegAB, had no influence on virulence of *B*. *pseudomallei*. These data differ from results of Mangalea et al. (2020) which shows that Δ*narX/ΔnarL* mutants were significantly less able to survive within macrophages [53]. Although we observed a slight decrease in intracellular replication in RAW 264.7 macrophages for the Δ*narL* strain, the effect did not pass our significance criteria. In line with this observation, mutants containing deletions in individual NarL- and FNR-dependent genes involved in the dissimilatory nitrate reduction (*narG*, BPSL2351, BPSL2355, *nosZ*), the anaerobic heme biosynthesis (*hemN*) and two cytochrome *bd* ubiquinol oxidase systems (*cydAB*) all retained a virulente phenotype. The lack of a virulence phenotype might result from partial redundancy and simultaneous downregulation of several of these genes and/or operons might be necessary to cause a significant loss in virulence. Together, the data suggest that denitrification *per se* is not required for pathogenicity in *B*. *pseudomallei*, but rather for the survival in the environment. Dance *et al*. (2000) proposed that *B*. *pseudomallei* used the nitrate reduction of fertilizers for their proliferation in rice fields [88]. In supported of this suggestion a study conducted in Thailand, showed an association of increased *B*. *pseudomallei* occurrence and nitrate concentrations in man-made wetlands [89] and a study from Australia showed that the usage of fertilizer rich on nitrate directly promote *B*. *pseudomallei* growth across different soil types as found in domestic gardens [90].

Why does loss of RegAB function leads to decreased virulence? Our experimental approach excludes nitrate respiration as major fitness factor during infection. In addition to *narL* and *fnr*, RegA transcriptionally activates several presently uncharacterized regulators of the Crp/FNR class and LysR family. Members of these groups of regulators coordinates many essential metabolic pathways in bacteria by responding to intracellular and exogenous signals, such as cAMP, oxidative and nitrosative stresses, carbon monoxide, 2-oxo-glutarate, temperature and/or intermediates of central metabolism [51,91–97]. Homologs of these proteins were shown to play a role in virulence in other bacteria e.g. Crp in *Yersinia pestis* [98]. Potential Crp-targets, as for example pentose phosphate metabolism, the methylcitric acid cycle, were upregulated in the Δ*regA* mutant. This suggests that RegAB could contribute to avoid an imbalances of the NAD+/NADH and NADP+/NADPH redox couples during anaerobiosis via transcriptional control by a Crp homolog. Thus, a more severe deregulation of the metabolisms might occur in a RegAB deficient strain, which is not evident if only a single downstream regulator or metabolic gene is affected.

## Conclusions

**Fig 8:**
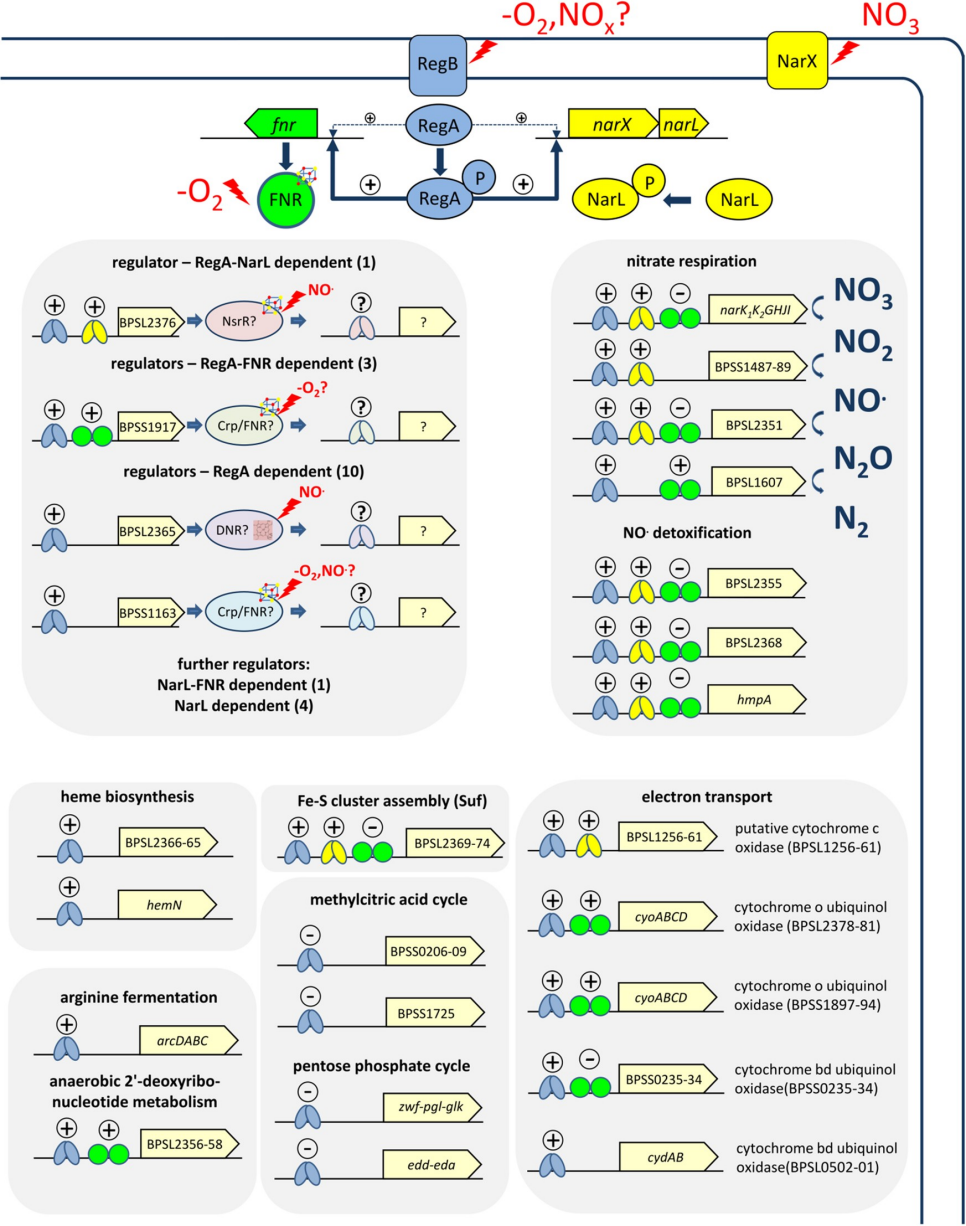
Preliminary regulatory model of gene expression during anaerobiosis in *B*. *pseudomallei*. Under normoxic conditions the RegAB TCS positively influences the gene expression of transcriptional regulators like the NarXL TCS and FNR as well as few other genes (not shown) on a low basis level. It ensured their presence and maintained their regulatory function under all growth conditions. Under anaerobic conditions (-O_2_), the RegB sensor kinase is activated and phosphorylates the RegA response regulator leading to a strong transcriptional activation of the NarXL TCS, FNR and further regulatory systems. Altogether their expression and simultaneous functional activation by the lowered redox potential and presence of nitrate (NO_3_^-^) and/or different intermediates of nitrate degradation (e.g. NOx, NO) drives the regulation of genes involved in nitrate respiration, NO detoxification, alternative electron transport chains, heme biosynthesis, arginine fermentation, Fe-S cluster assembly, anaerobic nucleotide metabolism, methylcitric acid cycle and the pentose phosphate cycle. Blue symbolizes the RegA response regulator, green the FNR regulator and yellow the NarL response regulator. Plus signs indicate a putative role as activator, minus signs as a putative repressor and the question mark indicates still unknown roles in the regulation of their respective genes and operons. small circle with plus sign–low induction, big circle with plus sign–high induction.

In summary, our transcriptomics and phenotypic data suggest a first model of anaerobic gene regulation in *B*. *pseudomallei* (Fig 8). Under normoxia the RegAB TCSTS remains mainly inactive. Under hypoxic conditions, the RegAB TCSTS activate the gene expression of anaerobic metabolism and additional regulators. In the presence of nitrate as TEA, nitrate and its metabolic degradation products further stimulate transcription/activation of regulator NarXL TCSTS, Dnr, NsrR, which are also regulated by RegAB, gearing the metabolism towards efficient nitrate respiration. The anaerobic metabolism is further fine-tuned by FNR, which is also controlled by RegAB but contrary to NarL, independent from nitrate for full activation. Thus, FNR might be particularly important to prepare the metabolism to the use of varying TEA by regulating *e*.*g*. diverse electron transport systems. However, even in the presence of oxygen a basal activity of RegAB and consequently transcription of *narXL* and *fnr* was observed. This would allow a rapid adaptation to oxygen limited conditions, placing RegAB as a master regulator at the cross-road of aerobic and anaerobic metabolism.

## Material and methods

### Ethics statement

All the animal experiments described in the present study were conducted in strict accordance with the recommendations in the Guide for the Care and Use of Laboratory Animals of the National Institutes of Health. All animal studies were conducted under a protocol approved by the Landesamt für Landwirtschaft, Lebensmittelsicherheit und Fischerei Mecklenburg-Vorpommern (LALLF M-V; 7221.3–1.1-044/05 and 7221.3–1.1-020/11). All efforts were made to minimize suffering and ensure the highest ethical and human standards.

### Bacterial strains, media, reagents, and growth conditions

The bacterial strains used in this study are listed in S1 Table. *B*. *pseudomallei* E8 was isolated from soil in the area surrounding Ubon Ratchathani, north-east Thailand [99]. Strains of *B*. *pseudomallei* were grown on Columbia agar or Luria-Bertani (LB) agar plates or LB broth under aerobic or anaerobic conditions. When appropriate, antibiotics were added at the following concentrations: 25 μg ml^-1^ chloramphenicol (Cm), 25 and 12.5 μg ml^-1^ tetracycline (Tc) for *E*. *coli* SM10(pOT182), 100 μg ml^-1^ streptomycin (Sm) and 50 μg ml^-1^ tetracycline for *B*. *pseudomallei* Tn*5* (pOT182) mutants, 100 μg ml^-1^ ampicillin (Ap) for *E*. *coli* DH5α (pTNS3) and 35 μg ml^-1^ kanamycin for *E*. *coli* HB101(pRK2013). For specific deletion mutagenesis, antibiotics were added at the following concentrations: *E*. *coli* S-17.1 λ pir 25 μg ml^-1^ tetracyclin or 30 μg ml^-1^ kanamycin (Km) and *B*. *pseudomallei* 3000 μg ml^-1^ zeocin (Zeo) and 125 μg ml^-1^ polymyxin B (Pm), 50 μg ml^-1^ tetracyclin and 125 μg ml^-1^ Pm, 500 μg ml^-1^ kanamycin and 125 μg ml^-1^ polymyxin B or solely 500 μg ml^-1^ kanamycin. All chemicals were obtained from Sigma-Aldrich unless stated otherwise. *B*. *pseudomallei* experiments were carried out in biosafety level 3 (BSL3) laboratories.

### *B*. *pseudomallei* Tn*5*-OT182 mutagenesis and anaerobic growth screening

The plasmids used in this study are listed in S1 Table. Genome-wide mutagenesis of *B*. *pseudomallei* strain E8 was performed as described previously [29] with Tn*5*-OT182 followed by an analysis of the mutants for their anaerobic growth ability in thioglyconate broth supplemented with 100 mM nitrate. The anaerobic growth was performed using the BD GasPak EZ anaerobe pouch system (USA). Mutants that exhibited strong growth arrests compared with the wild type strain were selected to determine the transposon insertion site as described previously [29]. In one mutant, the transposon inserted behind base 242 of the BPSL0201 locus. This mutant was selected for further analysis and termed *B*. *pseudomallei* Δ*regB* (ΔBPSL0201).

### Deletion of genes in *B*. *pseudomallei*

Gene deletions were performed using up- and downstream PCR fragments, which were both cloned into the formally described pBAKA-*tetRA* plasmid and which were generated with primers listed in S1 Table [100]. The up-and downstream fragments were digested with restriction enzymes as shown in S1 Table. The corresponding sites in the pBAKA-*tetRA* plasmid were used for insertion of the two fragments. The resulting up- and downstream fragments containing pBAKA-*tetRA* plasmids were transformed into *E*. *coli* S-17.1λpir by heat shock and then transferred conjugally by *E*. *coli* S-17.1λpir to *B*. *pseudomallei* on membrane filters obtained from overnight cultures. The filters were incubated at 37°C on nonselective LB agar for 8–18 h before transferring cells from the filters to LB agar containing Tc and Pm. Plates were incubated at 37°C for 2 days. To induce a second crossing over and delete the plasmid backbone, colonies were spread on M9 minimal agar plates containing 0.1% 4-chloro-phenylalanine. The deletions were verified by PCR using respective primers (S1 Table). The Tc^R^ isolates were cured from the Tc resistance cassette by Flipase(Flp)-FRT recombination. To this end, the pFlpe4 plasmid that encodes the Flp enzyme was transferred to the mutant isolates by conjugation with *E*. *coli* S-17.1λpir as described above. Filters were incubated at 37°C on nonselective LB agar for 8–18 h before spreading cells to LB agar containing Km and Pm. LB agar plates were incubated at 37°C for two days. Next, the resulting colonies were streaked on LB agar containing 0.2% rhamnose and incubated at 30°C for 1–2 days. Afterwards, colonies were tested for Tc sensitivity and Km resistance. Selected colonies were streaked on LB agar and incubated at 42°C for 2 days to cure them from the pFlpe4 plasmid. Finally, successful mutagenesis was verified by PCR using the upstreamfor and dwstreamrev primer pair. Positive isolates were stored at − 80°C in LB medium containing 20% glycerol until further use.

### Complementations of *B*. *pseudomallei* mutants

All mutant strains (deletion mutants and Tn*5* mutant) were complemented with the mini-Tn7 system as described by Choi *et al*. (2005) [101]. Briefly, respective genes were amplified by PCR using primers creating fragments containing up to 471 bp upstream of the predicted start codon (S1 Table). The resulting fragments were digested with respective restriction enzymes and cloned into the pUC18T-mini-Tn7-Zeo-*loxP* or pUC18TminiTn7-Km-FRT vector using the corresponding restriction sites. The resulting vectors were transformed into *E*. *coli* DH5α by heat shock and conjugally transferred by tetraparental mating using the additional *E*. *coli* helper strain HB101 (pRK2013), DH5α-pTNS3 to the *B*. *pseudomallei* recipient mutant strain. Bacteria were selected on LB agar plates containing Km and Pm or Zeo and Pm. The mini-Tn7-elements were transposed to either one of three attTn7 sites downstream of the glutamine-6-phosphate synthases *glmS1*, *glmS2* and *glmS3*. Successful insertion at the *glmS1*, *glmS2*, and *glmS3* linked insertion sites were verified using the primer TnL7 (ATTAGCTTACGACGCTACACCC) and one of the site specific primers BPGLMS1 (GAGGAGTGGGCGTCGATCAAC), BPGLMS2 (ACACGACGCAAGAGCGGAATC), and BPGLMS3 (CGGACAGGTTCGCGCCATGC). All isolates were stored at − 80°C in LB medium containing 20% glycerol until further use.

### Detection of nitrate and nitrite

The concentrations of nitrate and nitrite were determined in the supernatant of aerobically and anaerobically grown cells. At the respective times, the cells were separated from the supernatant by centrifugation (10.000 x g, 2°C, 5 min), filtrated (0.20 μm, Sarstedt), and the amounts of the respective metabolites were measured by using the test combinations nitrate and nitrite (R-Biopharm AG, Germany) according to the manufacturer’s instructions.

### RNA extraction

For microarray experiments, total RNA was extracted from strains grown in LB medium or LB medium supplemented with 50 mM nitrate. For anaerobic conditions, the strains were first grown under aerobic conditions in 70 ml LB in 250-ml Erlenmeyer flasks under vigorous agitation at 37°C to an optical density at 650 nm (OD_650nm_) of 0.5. Afterwards cells were shifted to anaerobic growth by transferring the cultures to Falcon tubes (50 ml) which were completely filled and incubated under vigorous agitation at 37°C for at least 30 min. Anaerobic conditions were verified by using 0.001% resazurin as a redox indicator [84]. Microaerophilic and anaerobic conditions were observed at 10 min after the shift. Fifty milliliters of cell suspension were harvested 30 min after shift and centrifuged at 2°C at 10.000 x g for 5 min. Cell pellets were quickly resuspended in 1 ml of Trizol Reagent (Invitrogen). After the RNA extraction procedure according to manufacturer’s instructions, the integrity of the RNA was assessed by agarose gel electrophoresis and tested for the absence of DNA contamination by PCR.

### *B*. *pseudomallei* tiling microarrays and expression profiling

For transcriptomics, we used high-density tiling arrays fabricated by Roche NimbleGen (Roche NimbleGen, United States) based on the *B*. *pseudomallei* K96243 reference genome [25]. From each strain three independent RNA preparations were reverse transcribed and Cy3 or Cy5 labeled. To obtain DNA-free RNA, 100 μg total RNA per sample were treated with DNA-free Kit from Ambion (United States) according to the manufacturer’s instructions. RNA was precipitated with ethanol and resuspended in nuclease free water. Afterwards, 10 μg per sample were subjected to ribosomal RNA depletion using the MICROBExpress kit from Ambion (United States) according to the manufacturer’s instructions. Next, 2 μg of purified mRNA was used for cDNA synthesis utilizing Superscript II Reverse Transcriptase (Life Technologies). Finally, 1.5 μg of obtained cDNA were labeled according to the manufacturer’s instructions (ULS arrayCGH Labeling Kit (Leica Microsystems). Hybridization and microarray scanning were processed as described before [102,103]. The microarray images were analyzed by Roche NimbleScan software (Roche NimbleGen, United States) and obtained signals were normalized using Robust Multi-array Average method (RMA) (Roche NimbleGen, United States). Only those probes downstream of the annotated translational start site were considered for estimating the fold change of gene expression. For this, Cy3/Cy5 (wild type / respective mutant Δ*regA*, Δ*narL* or Δ*fnr*) ratios were first transformed using the binary logarithm and analyzed using two-way ANOVA (factor 1: wild type, Δ*regA*, Δ*narL*, Δ*fnr*; factor 2: aerobic, anaerobic) using MeV v4.8.1 [104]. Values with a *p*-value < 0.01 were considered as significant. Further, only genes showing in all three biological replicates an at least two-fold induction/repression (log_2_ < -1 or log_2_ > 1) between wild type and one of the mutants were considered as differentially expressed. The normalized signals from all three biological replicates were averaged to obtain a single value. Complete microarray dataset has been deposited in the Gene Expression Omnibus (GEO) under accession number GSE159521.

### Quantitative real-time PCR

For quantitative real-time PCR (qRT-PCR) analysis, *B*. *pseudomallei* wild type and mutant strains were cultivated and harvested as described above. Total RNA was extracted by the Trizol method, as described by the manufacturer (Invitrogen). DNA was removed using DNase I (Thermo Fisher Scientific) and its absence was confirmed by PCR. qRT-PCR was performed using 5 μg of total RNA, 200 U of Superscript II Reverse Transcriptase (Invitrogen) and 500 ng of random primers, following the manufacturer’s instructions. Quantitative PCR amplification of the resulting cDNA was performed with Platinum SYBR Green (Applied Biosystems) and gene-specific primers (S1 Table). The primers were designed using the OligoPerfect primer designing tool from Invitrogen (http://www.invitrogen.com). All qRT PCRs were run with three biological replicates. Results were normalized using the 23S rRNA gene displaying constant expression levels as endogenous control as previously described [105]. Relative expression levels were calculated using the 2^−ΔΔCT^ method [106].

### Infection assays of HeLa cells and RAW264.7 macrophages

RAW 264.7 macrophages were cultivated in Dulbecco’s Modified Eagle Medium (DMEM, Thermo Fisher Scientific, United States), HeLa cells in Mimimal Essential Medium (MEM, Biochrom) and both cell lines were supplemented with 10% fetal calf serum (FCS, Capricorn GmbH, Germany). In addition, the MEM medium was further supplemented with sodium pyruvate (1 mM), non-essential amino acids (NEA, 1%) and glutamine (1 mM). Twenty-four hours prior to infection, cells were seeded in 48-well plates at 9 × 10^4^ cells per well. *B*. *pseudomallei* wild type, mutant or the complemented mutant strains were added to the macrophages at MOI 5 and MOI 10 with HeLa-T cells followed by low speed centrifugation at 120 x g for 4 min at RT to initiate infection. For the adhesion assay, 30 min post-infection the cells were washed twice with sterile PBS to remove unbound bacteria. Then macrophages with the adhered bacteria were lysed with Tergitol (1%) and plated on LB agar plates to determine the colony forming units (CFU). For the invasion assay, 30 min post-infection the cells were washed three times with sterile PBS to remove unbound bacteria. Washed macrophages and HeLa-T cells were cultivated for further 30 min at 37°C in fresh medium containing 750 μg ml^−1^ kanamycin to kill remaining extra cellular bacteria. Afterwards cells were lysed with Tergitol (1%) and plated on LB agar plates to determine the CFU. For the intracellular replication assays, 30 min post-infection the cells were washed three times with sterile PBS to remove unbound bacteria. After it (time 0; 0 h), cells were incubated in fresh medium containing 750 μg ml^−1^ kanamycin (plus 3 μg tetracyclin for complemented mutant strains) and infected cells were incubated for further 6 and 24 h under aerobic conditions. To gain anaerobic conditions, cells were handled as described above but finally incubated using the BD GasPak EZ anaerobe pouch system (Becton Dickinson, USA). At the respective time points, the numbers of CFU were determined per well by plating serial dilutions of the Tergitol (1%)—lysed cells on LB agar plates.

### Murine infection model

Female 8- to 12-week-old BALB/c mice were obtained from Charles River Wiga Deutschland GmbH (Sulzfeld, Germany). All *in vivo* studies were approved by the local authority. Animals were maintained under specific pathogen-free conditions and were provided with food and water *ad libitum*. Bacteria were grown for 16 h on LB agar supplemented with 5% sheep blood and adjusted to an OD_650_ of 0.25 in sterile PBS. Prior to intranasal (*i*.*n*.) application mice were anesthetized with a mixture of ketamine hydrochloride and xylazine hydrochloride. Thirty microliters of the bacterial suspension were inoculated into both nostrils of an animal (15 μl per nostril). For intravenous applications (*i*.*v*.), mice were fixed in plastic tubes and the bacterial suspension of 0.2 ml was injected into the lateral tail vein. Animals were monitored daily for signs of disease and mortality. To enumerate bacteria in the spleen, liver, and lungs, the organs were aseptically removed 48 h after infection, homogenized in 0.5–1 ml sterile PBS containing 0.5% Tergitol and 1% BSA. The suspensions were diluted and plated on Ashdown agar plates [107] to determine the number of CFUs per organ.

### Sequence analysis of RegA and RegB

Protein sequences related to the *B*. *pseudomallei* RegA RR and RegB SK were extracted from EggNOG orthologues groups ENOG4108UYB (RegA) and ENOG4105TX3 (RegB). If a species was represented by multiple strains, a single representative sequence was selected. In addition, RegA and RegB paralogs were included if present within the analyzed species. The final analysis involved 169 amino acid sequences for each protein family. RegA and RegB sequences were aligned using ClustalO [108] and the evolutionary history inferred by using the Maximum Likelihood method based on the Le & Gascuel model as implemented in MEGA [109,110]. Different evolutionary models for phylogenetic inference were tested to identify the model yielding the best maximum likelihood values. For both proteins a discrete Gamma distribution (5 categories (RegA = +G, parameter = 0.5850; RegB = +G, parameter = 0.9540) was chosen. For RegB some sites were allowed to be evolutionarily invariable (+I, 1.18% sites). All positions containing gaps and missing data were eliminated. There were a total of 143 (RegA) and 211 (RegB) positions in the final dataset. Bootstrapped consensus trees were inferred from 500 replicates.

BLAST (Basic Local Alignment Search Tool) analyses of RegA (BPSL0202) and RegB (BPSL0201) amino acid sequences were done using ProteinBLAST from NCBI (https://blast.ncbi.nlm.nih.gov/Blast.cgi?PROGRAM=blastp&PAGE_TYPE=BlastSearch&LINK_LOC=blasthome) and multiple sequence alignments of RegA/RegB protein sequences of different species were done with the CLUSTAL 2.1 tool (https://www.genome.jp/tools-bin/clustalw).

### Statistical analysis and used software

Data are expressed as mean values ± standard error of the mean (SEM) and analyzed using Student’s t-test, one-way ANOVA with Bonferroni multiple comparisons posttest or two-way ANOVA with Bonferroni multiple comparisons posttest as indicated in the figure legends. Survival data of the mice were analyzed using the Kaplan–Meier method. Unless otherwise indicated the *p*-value of <0.05 was considered as statistically significant. All data were processed in Excel 2010 (Microsoft Cooperation), Prism software (version 5.02; GraphPad Software), Paver (DECODON GmbH) and/or MeV v4.8.1 software [104].

## Supporting information

S1 Fig Growth of *B*. *pseudomallei* wild type (wt), the Tn*5* transposon mutant (Tn*5*Δ*regB*) and the complemented transposon mutants (ComTn*5*Δ*regB*::*regB* and ComTn5Δ*regB*::*regB-regA*) (S1A), genetic organization of the *regAB* region (S1B) and mortality curves of BALB/c mice infected with the same strains (S1C). S1A: Strains were cultivated in microtiter plates in thioglyconate broth with 100 mM nitrate and incubated at 37°C for 24 h under anaerobic conditions. After 24 hours the growth was photographically monitored. Shown is one representative out of three independently performed experiments. S1B: Localization of the *regB* (BPSL0201) and *regA* (BPSL0202) genes on chromosome I and the Tn*5* insertion site (black triangle) in the Δ*regB* mutant. The Tn*5* insertion in BPSL0201 occurred after nucleotide 242. Annotation of the neighboring genes: BPSL0200, acetylglutamate kinase [EC:2.7.2.8]; BPSL0203, ATP-dependent HslUV protease ATP-binding subunit HslU. S1C: Mice (n = 10) were intravenously infected with low doses (200 CFU) of all strains and mortality curves were determined. Shown is one representative out of three independently performed experiments. Curves were compared by using the log rank Kaplan-Meier test (*p* = 0.0175).
(TIF)

S2 Fig Alignment of RegB sensor kinases (A) and RegA response regulators (B) of *B*. *pseudomallei* with different bacterial species. A: Transmembrane domains are indicated as regions TM1 through TM6 and marked as gray boxes. Further denoted are highly conserved domains as the ubiquinone binding pocket (blue), the H-box embedded in the dimerization domain (green) and the redox box (red). Stars denote highly conserved amino acids. -B: The input domain contains a conserved phosphate-accepting aspartate denoted by red colour. Stars denote highly conserved amino acids. Shown are sequences of RegB and RegA TCSs of the following bacterial species: *Burkholderia pseudomallei* (BPSL201/BPSL0202), *Sinorhizobium meliloti* 1021 (SMc02585/SMc2584), *Rhodobacter capsulatus* (RCAP_rcc1026/RCAP_rcc1025; RCAP_rcc00043/RCAP_rcc00045), *Pseudomonas aeruginosa* (PA4494/PA4493), *Caulobacter crescentus* (CC_1768/CC_1767; CC_0248/CC_0247), *Brucella suis* (BSSP2_I0131/BSSP2_I0134), *Brucella melitensis* (DK63_1673/DK63_1676), *Agrobacterium tumefaciens* (Ach5_00510/Ach5_00500).
(TIF)

S3 Fig Bootstrap consensus tree inferring molecular phylogenetic of RegA and RegB. Maximum likelihood analysis was used to infer the evolutionary relationship for RegA (A) and RegB (B). Branches corresponding to partitions reproduced in less than 50% bootstrap replicates are collapsed. Well separated sequence clusters are highlighted by color. Taxonomic groups are indicated by symbols: open circle = alpha, open square = beta, black circle = gamma, open triangle = delta, open diamond = epsilon, black square = zeta proteobacteria, black triangle = Acidobacteria, black diamond = Planctomycetacia and open triangle tip down = Verrucomicrobia. To distinguish paralogues sequences, they are displayed in blue and red. In addition, blue asterisks mark group 1 and red asterisks group 2 RegA or RegB homologs concerning to Elsen et al. 2004 [15].
(TIF)

S4 Fig Growth curves of *B*. *pseudomallei* wild type, Δ*fnr* and Δ*narL* mutants and their respective complemented mutant strains cultivated under aerobic (A, B) and anaerobic (C, D, E) conditions in LB medium without (A, C) or with (B, D, E) 50 mM nitrate. Shown are mean values of three independent experiments. Error bars indicate standard error of the mean (SEM).
(TIF)

S5 Fig Growth curves of *B*. *pseudomallei* wild type, different mutants and their respective complemented mutant strains cultivated under aerobic (A, B) and anaerobic (C, D, E) conditions in LB medium without (A, C) or with (B, D, E) 50 mM nitrate. Shown are mean values of three independent experiments. Error bars indicate standard error of the mean (SEM).
(TIF)

S6 Fig Determination of nitrate and nitrite in supernatants of anaerobically grown *B*. *pseudomallei* mutants and their respective complemented strains. Cells were anaerobically cultivated in LB medium supplemented with 50 mM nitrate. Supernatants were harvested and concentration of nitrate and nitrite were measured as described in Material and Methods. Shown are mean values of three independent experiments. Error bars indicate standard error of the mean (SEM).
(TIF)

S7 Fig Replication of *B*. *pseudomallei* mutant strains in RAW264.7 macrophages. Wild type and mutants were cultivated aerobically on LB agar for 16 hours at 37°C. Then, cells were diluted in PBS and used at MOI 2 with RAW264.7 macrophages for infections. Infected macrophages were incubated at 37°C for up to 24 h under aerobic or anaerobic conditions. At indicated time points (0, 6 and 24 hours), the infected RAW264.7 cells were lysed and dilutions were plated on LB agar plates. After 48 h of growth at 37°C colonies of *B*. *pseudomallei* were counted on the plates and CFU/well was calculated. The percentage of replication represented the number of intracellular bacteria relative to initial count at time point 0 h. Shown are mean values of three independent experiments. Error bars indicate standard error of the mean (SEM).
(TIF)

S8 Fig Mortality curves of BALB/c mice infected with the *B*. *pseudomallei* wild type and different mutant strains. Mice (n = 4) were intranasally infected with low dose (100 CFU) of all strains (E8: 90 CFU; ΔBPSL1607: 68 CFU; ΔBPSL2646: 83 CFU; ΔBPSS0235: 78 CFU; ΔBPSS0031: 70 CFU; ΔBPSL2355: 45 CFU; ΔBPSL2351: 53 CFU; ΔBPSS1159: 100 CFU; ΔBPSL2309: 80 CFU; ΔBPSL2314: 85 CFU; ΔBPSL0502: 123 CFU). Curves were compared by using the log rank Kaplan- Meier test. wild type–black line; red line—respective mutant strain.
(TIF)

S9 Fig Quantitative RT-PCR (qRT PCR) validation of tiling microarray results of selected genes. Gene expression differences in different mutant strains (Δ*regA*, Δ*narL* and Δ*fnr*) measured by qRT-PCR are represented as fold changes relative to the *B*. *pseudomallei* wild type strain E8 (wt). Data from qRT PCR experiments were normalized using the 23S rRNA gene as internal control. Mean values of three independent experiments are displayed. Error bars indicate standard error of the mean (SEM). BPSS0031—anaerobic growth regulatory protein FNR, BPSL0202—response regulator protein RegA, BPSS0368—TonB-like transport protein, BPSL0502—cytochrome d ubiquinol oxidase subunit I, BPSS0518—type VI secretion system secreted protein HcpI, BPSS1163—fumarate/nitrate reduction family regulatory protein, BPSL1259—putative cytochrome c oxidase subunit II related protein, BPSS1522—two-component response regulator, BPSS1524—intercellular spread protein, BPSS1529—membrane antigen, BPSL1607—nitrous-oxide reductase precurser, BPSS1613—type III secretion protein, BPSS1897—cytochrome o ubiquinol oxidase subunit II, BPSS1917—crp-family transcriptional regulator, BPSL2307—nitrite/nitrate transporter, BPSL2309—respiratory nitrate reductase alpha chain, BPSL2314—putative response regulator protein, BPSL2351—nitric oxide reductase subunit B, BPSL2356—anaerobic ribonucleoside triphosphate reductase, BPSL2363—putative 2-nitropropane dioxygenase, BPSL2369—cysteine desulfurase activator complex subunit SufB, BPSL2377—cation-binding hemerythrin HHE family protein, BPSL2378 3—ubiquinol oxidase polypeptide II precursor, BPSL2646—coproporphyrinogen III oxidase, BPSL2840 –flavohemoprotein Hmp.
(PDF)

S10 Fig Growth and nitrite consumption of *B*. *pseudomallei* wild type, Δ*regA* mutant and complemented mutant strain ComplΔ*regA* in LB medium with nitrite. **A**: Cells were cultivated under anaerobic conditions in LB medium supplemented with 5 mM nitrite at 37°C and 140 rpm over a time period of 48 hours. Shown are mean values of three independent experiments. Error bars indicate standard error of the mean (SEM). **B**: Supernatants were harvested and concentrations of nitrite were measured as described in Material and Methods. Shown are mean values of three independent experiments. Error bars indicate standard error of the mean (SEM).
(TIF)

S1 Table Table of used bacterial strains, plasmids and primers. (XLSX)

S2 Table Expression analysis of all significantly regulated RegA-, NarL- and FNR-dependent genes upon anaerobic conditions w/ and w/o nitrate. (XLSX)

S3 Table Results of the transposon screening showing Tn*5* mutants with severely disturbed growth under anaerobic condition with nitrate as TEA. (XLSX)
