# Supplementary figures and images for: RegAB Homolog of Burkholderia pseudomallei is the Master Regulator of Redox Control and involved in Virulence
Source: PLoS Pathog. 2021 May 28;17(5):e1009604. doi: 10.1371/journal.ppat.1009604 (PMC8191878; doi:10.1371/journal.ppat.1009604)

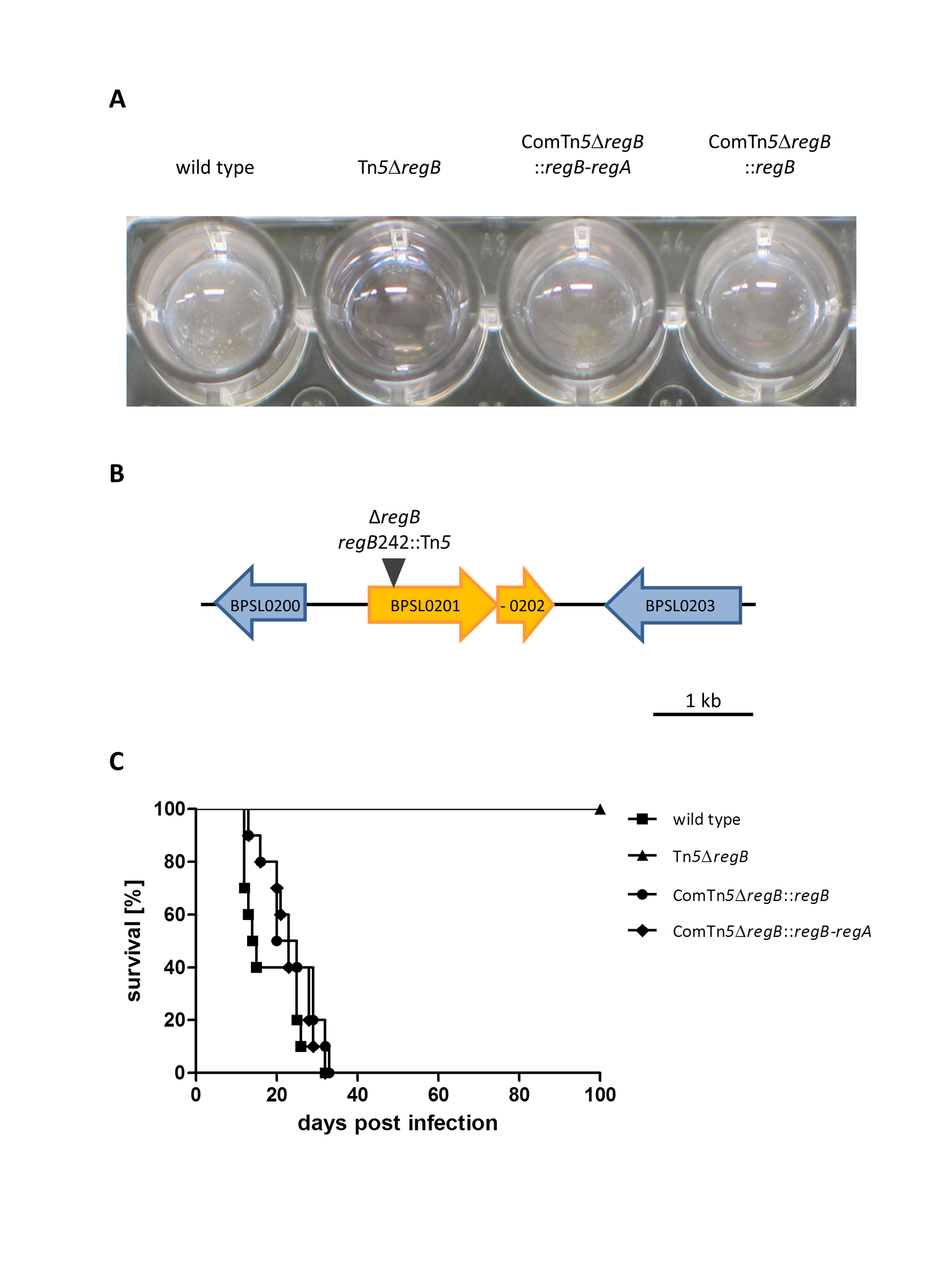

Supplement: S1 Fig — Growth of B. pseudomallei wild type (wt), the Tn5 transposon mutant (Tn5ΔregB) and the complemented transposon mutants (ComTn5ΔregB::regB and ComTn5ΔregB::regB-regA) (S1A), genetic organization of the regAB region (S1B) and mortality curves of BALB/c mice infected with the same strains (S1C). S1A: Strains were cultivated in microtiter plates in thioglyconate broth with 100 mM nitrate and incubated at 37°C for 24 h under anaerobic conditions. After 24 hours the growth was photographically monitored. Shown is one representative out of three independently performed experiments. S1B: Localization of the regB (BPSL0201) and regA (BPSL0202) genes on chromosome I and the Tn5 insertion site (black triangle) in the ΔregB mutant. The Tn5 insertion in BPSL0201 occurred after nucleotide 242. Annotation of the neighboring genes: BPSL0200, acetylglutamate kinase [EC:2.7.2.8]; BPSL0203, ATP-dependent HslUV protease ATP-binding subunit HslU. S1C: Mice (n = 10) were intravenously infected with low doses (200 CFU) of all strains and mortality curves were determined. Shown is one representative out of three independently performed experiments. Curves were compared by using the log rank Kaplan-Meier test (p = 0.0175). (TIF) [file ppat.1009604.s001.tif]

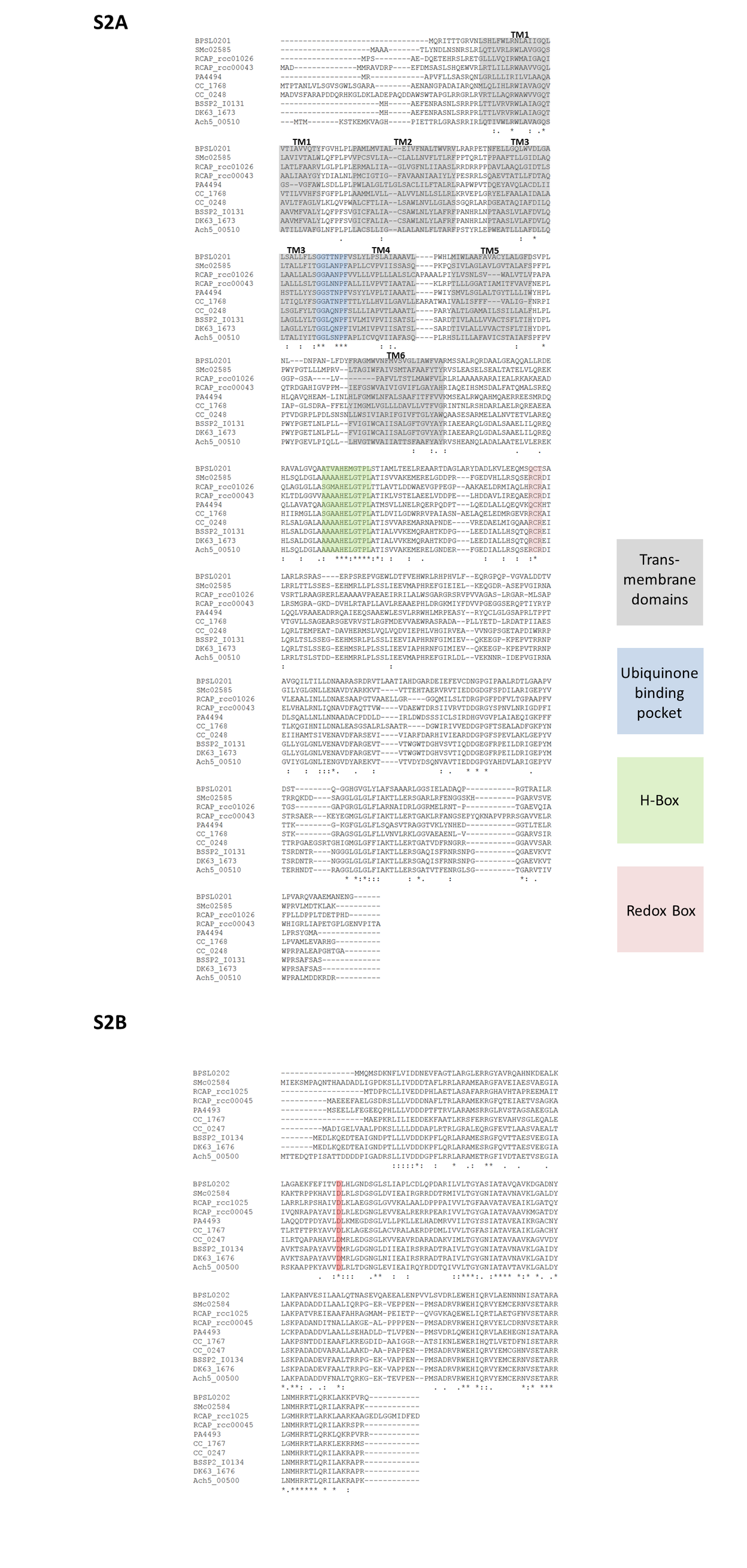

Supplement: S2 Fig — Alignment of RegB sensor kinases (A) and RegA response regulators (B) of B. pseudomallei with different bacterial species. A: Transmembrane domains are indicated as regions TM1 through TM6 and marked as gray boxes. Further denoted are highly conserved domains as the ubiquinone binding pocket (blue), the H-box embedded in the dimerization domain (green) and the redox box (red). Stars denote highly conserved amino acids. -B: The input domain contains a conserved phosphate-accepting aspartate denoted by red colour. Stars denote highly conserved amino acids. Shown are sequences of RegB and RegA TCSs of the following bacterial species: Burkholderia pseudomallei (BPSL201/BPSL0202), Sinorhizobium meliloti 1021 (SMc02585/SMc2584), Rhodobacter capsulatus (RCAP_rcc1026/RCAP_rcc1025; RCAP_rcc00043/RCAP_rcc00045), Pseudomonas aeruginosa (PA4494/PA4493), Caulobacter crescentus (CC_1768/CC_1767; CC_0248/CC_0247), Brucella suis (BSSP2_I0131/BSSP2_I0134), Brucella melitensis (DK63_1673/DK63_1676), Agrobacterium tumefaciens (Ach5_00510/Ach5_00500). (TIF) [file ppat.1009604.s002.tif]

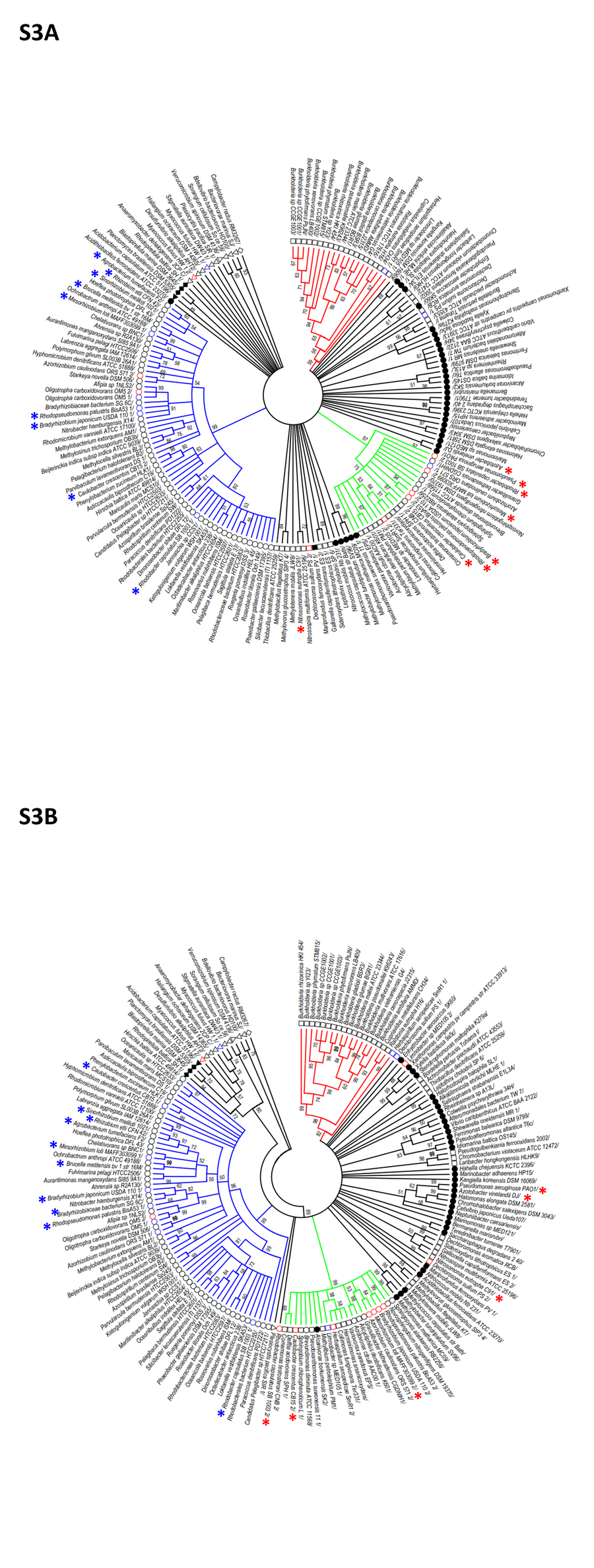

Supplement: S3 Fig — Maximum likelihood analysis was used to infer the evolutionary relationship for RegA (A) and RegB (B). Branches corresponding to partitions reproduced in less than 50% bootstrap replicates are collapsed. Well separated sequence clusters are highlighted by color. Taxonomic groups are indicated by symbols: open circle = alpha, open square = beta, black circle = gamma, open triangle = delta, open diamond = epsilon, black square = zeta proteobacteria, black triangle = Acidobacteria, black diamond = Planctomycetacia and open triangle tip down = Verrucomicrobia. To distinguish paralogues sequences, they are displayed in blue and red. In addition, blue asterisks mark group 1 and red asterisks group 2 RegA or RegB homologs concerning to Elsen et al. 2004 [15]. (TIF) [file ppat.1009604.s003.tif]

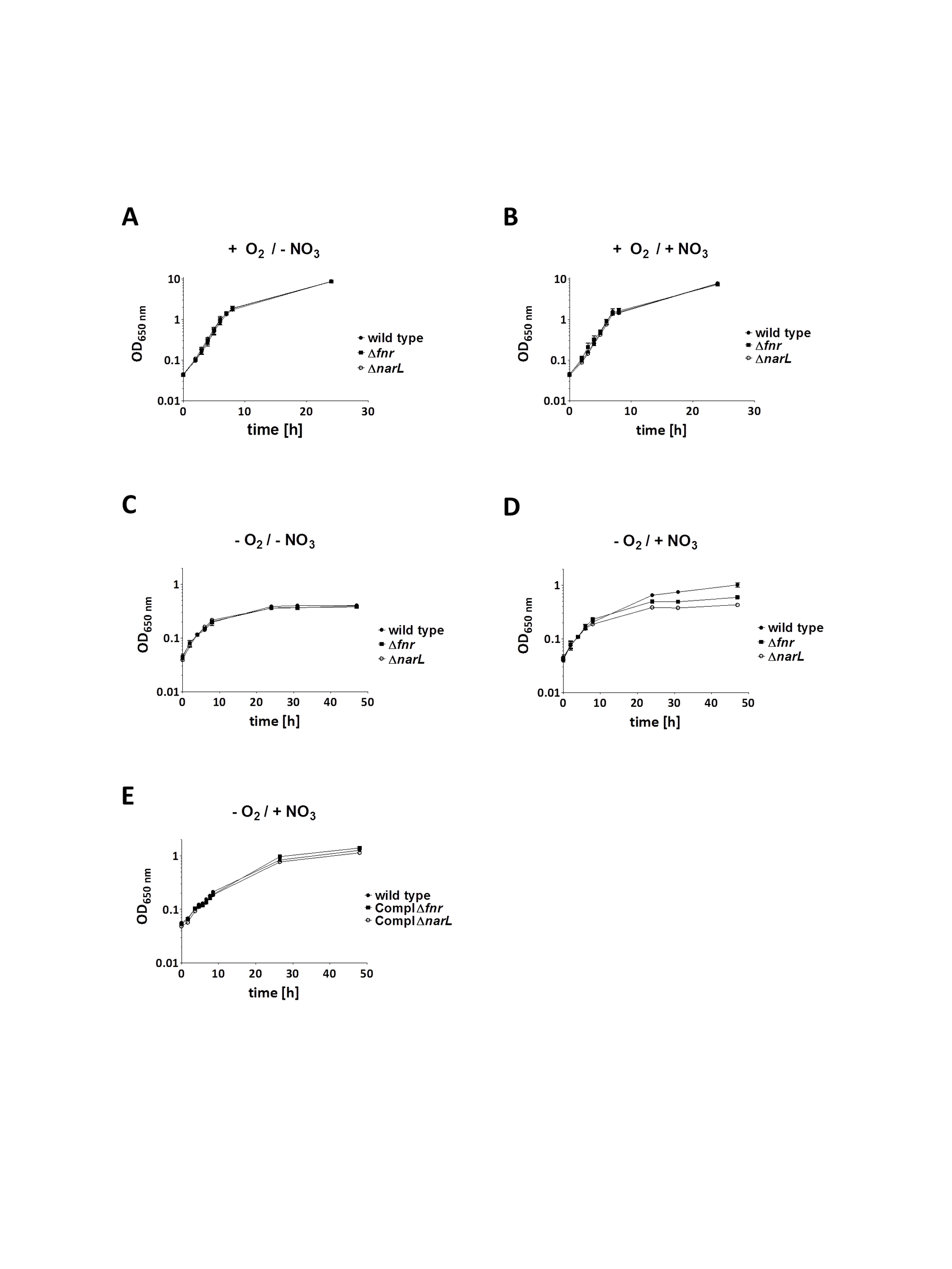

Supplement: S4 Fig — Growth curves of B. pseudomallei wild type, Δfnr and ΔnarL mutants and their respective complemented mutant strains cultivated under aerobic (A, B) and anaerobic (C, D, E) conditions in LB medium without (A, C) or with (B, D, E) 50 mM nitrate. Shown are mean values of three independent experiments. Error bars indicate standard error of the mean (SEM). (TIF) [file ppat.1009604.s004.tif]

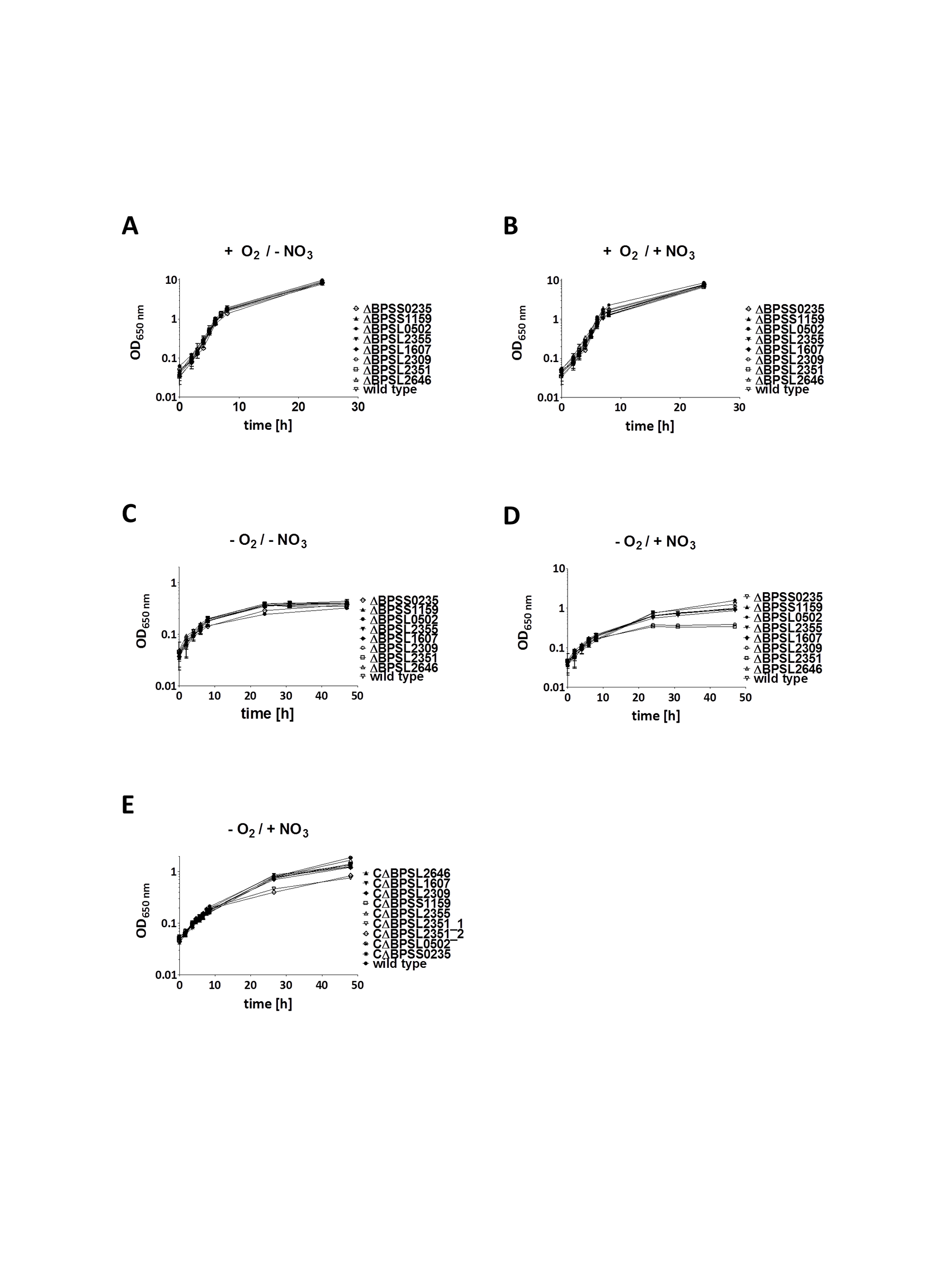

Supplement: S5 Fig — Growth curves of B. pseudomallei wild type, different mutants and their respective complemented mutant strains cultivated under aerobic (A, B) and anaerobic (C, D, E) conditions in LB medium without (A, C) or with (B, D, E) 50 mM nitrate. Shown are mean values of three independent experiments. Error bars indicate standard error of the mean (SEM). (TIF) [file ppat.1009604.s005.tif]

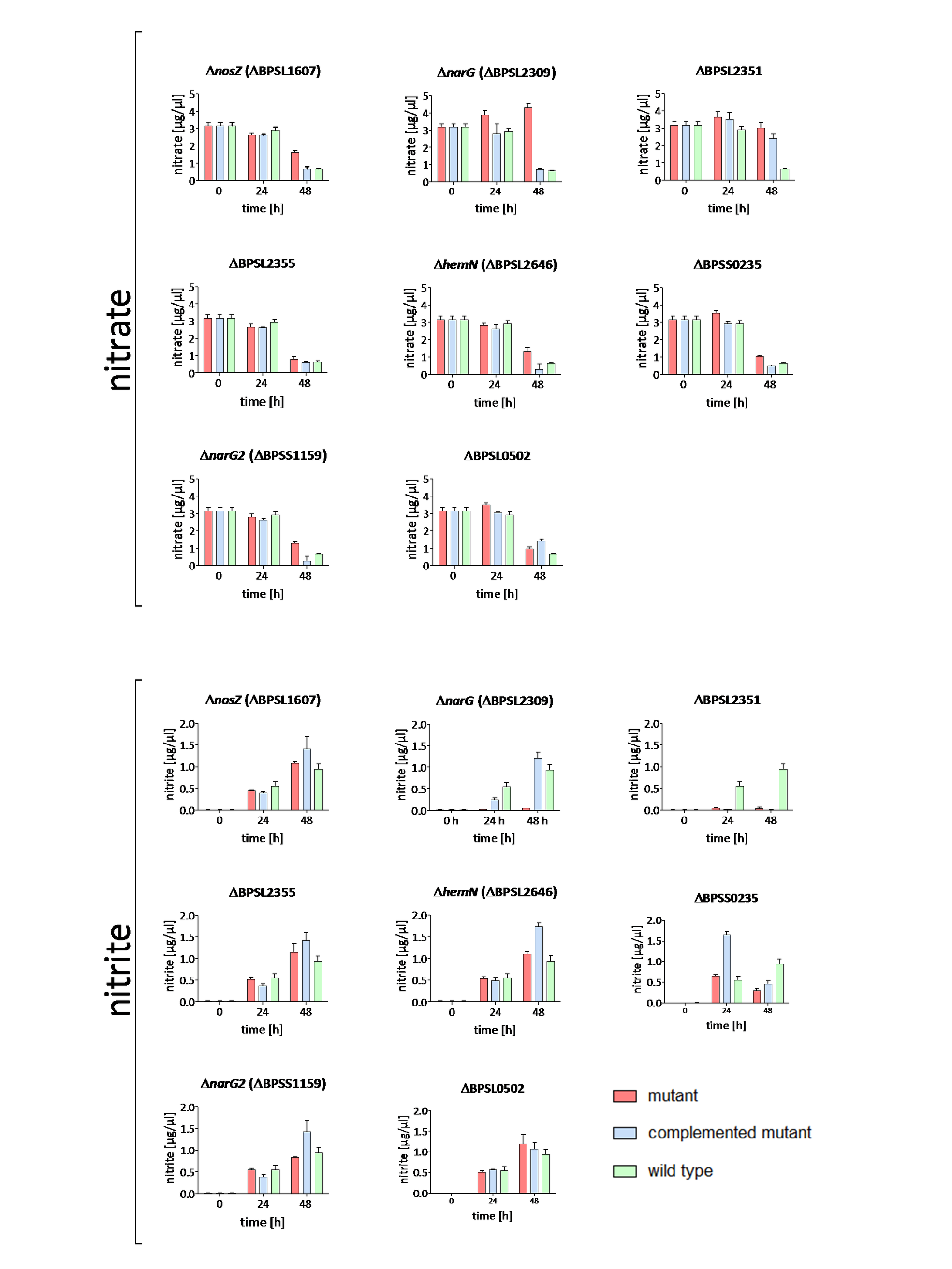

Supplement: S6 Fig — Cells were anaerobically cultivated in LB medium supplemented with 50 mM nitrate. Supernatants were harvested and concentration of nitrate and nitrite were measured as described in Material and Methods. Shown are mean values of three independent experiments. Error bars indicate standard error of the mean (SEM). (TIF) [file ppat.1009604.s006.tif]

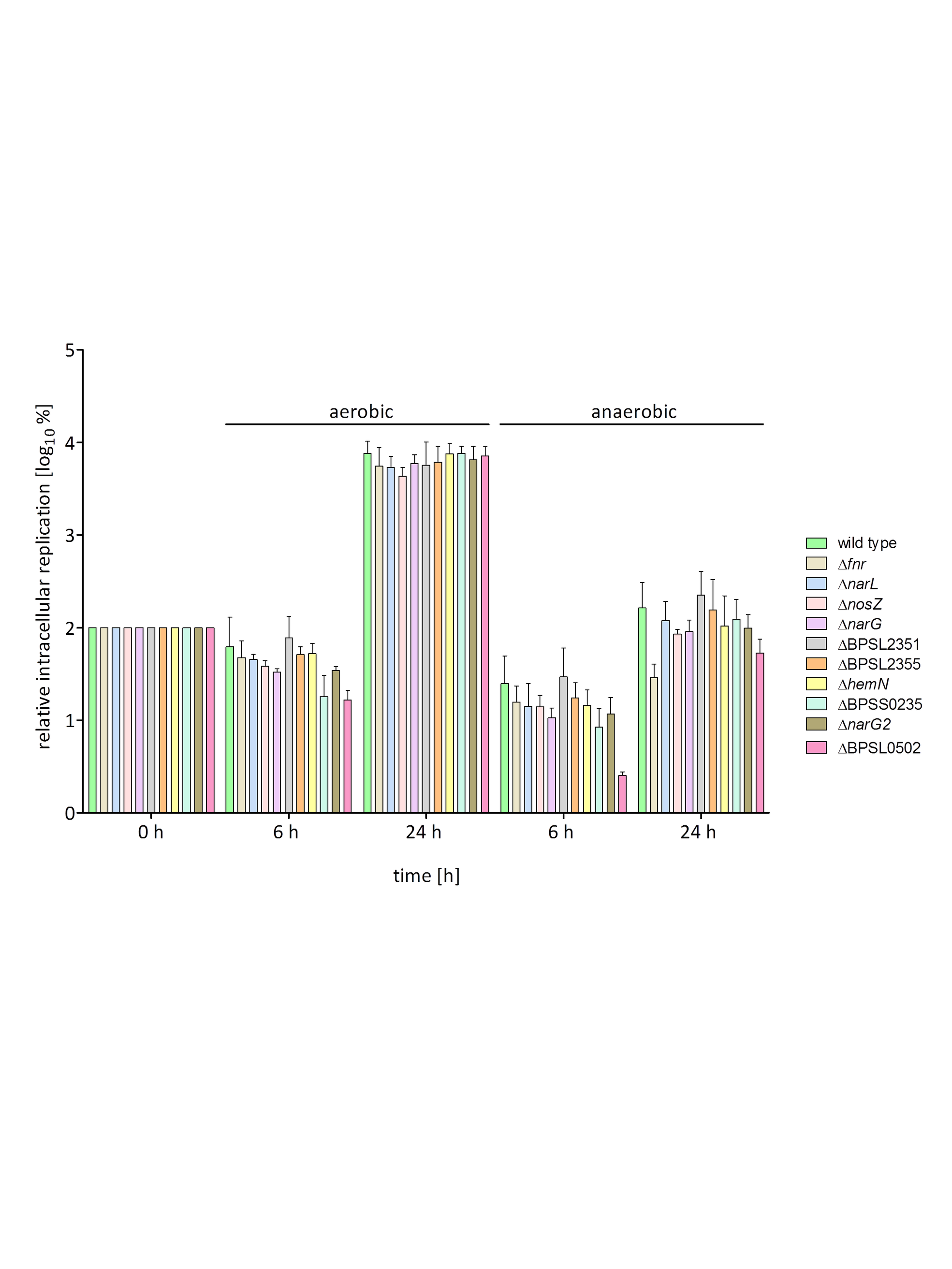

Supplement: S7 Fig — Wild type and mutants were cultivated aerobically on LB agar for 16 hours at 37°C. Then, cells were diluted in PBS and used at MOI 2 with RAW264.7 macrophages for infections. Infected macrophages were incubated at 37°C for up to 24 h under aerobic or anaerobic conditions. At indicated time points (0, 6 and 24 hours), the infected RAW264.7 cells were lysed and dilutions were plated on LB agar plates. After 48 h of growth at 37°C colonies of B. pseudomallei were counted on the plates and CFU/well was calculated. The percentage of replication represented the number of intracellular bacteria relative to initial count at time point 0 h. Shown are mean values of three independent experiments. Error bars indicate standard error of the mean (SEM). (TIF) [file ppat.1009604.s007.tif]

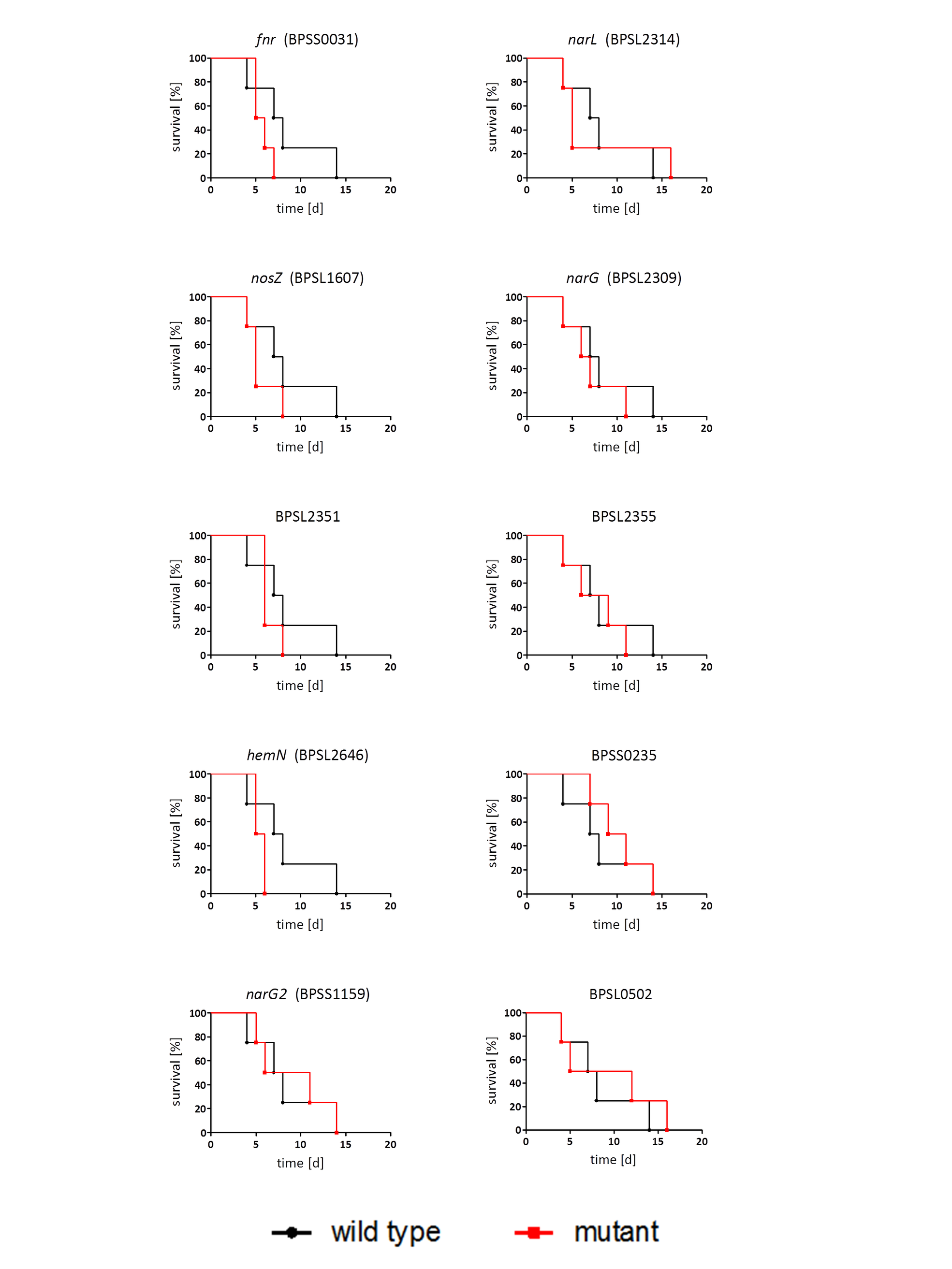

Supplement: S8 Fig — Mice (n = 4) were intranasally infected with low dose (100 CFU) of all strains (E8: 90 CFU; ΔBPSL1607: 68 CFU; ΔBPSL2646: 83 CFU; ΔBPSS0235: 78 CFU; ΔBPSS0031: 70 CFU; ΔBPSL2355: 45 CFU; ΔBPSL2351: 53 CFU; ΔBPSS1159: 100 CFU; ΔBPSL2309: 80 CFU; ΔBPSL2314: 85 CFU; ΔBPSL0502: 123 CFU). Curves were compared by using the log rank Kaplan- Meier test. wild type–black line; red line—respective mutant strain. (TIF) [file ppat.1009604.s008.tif]

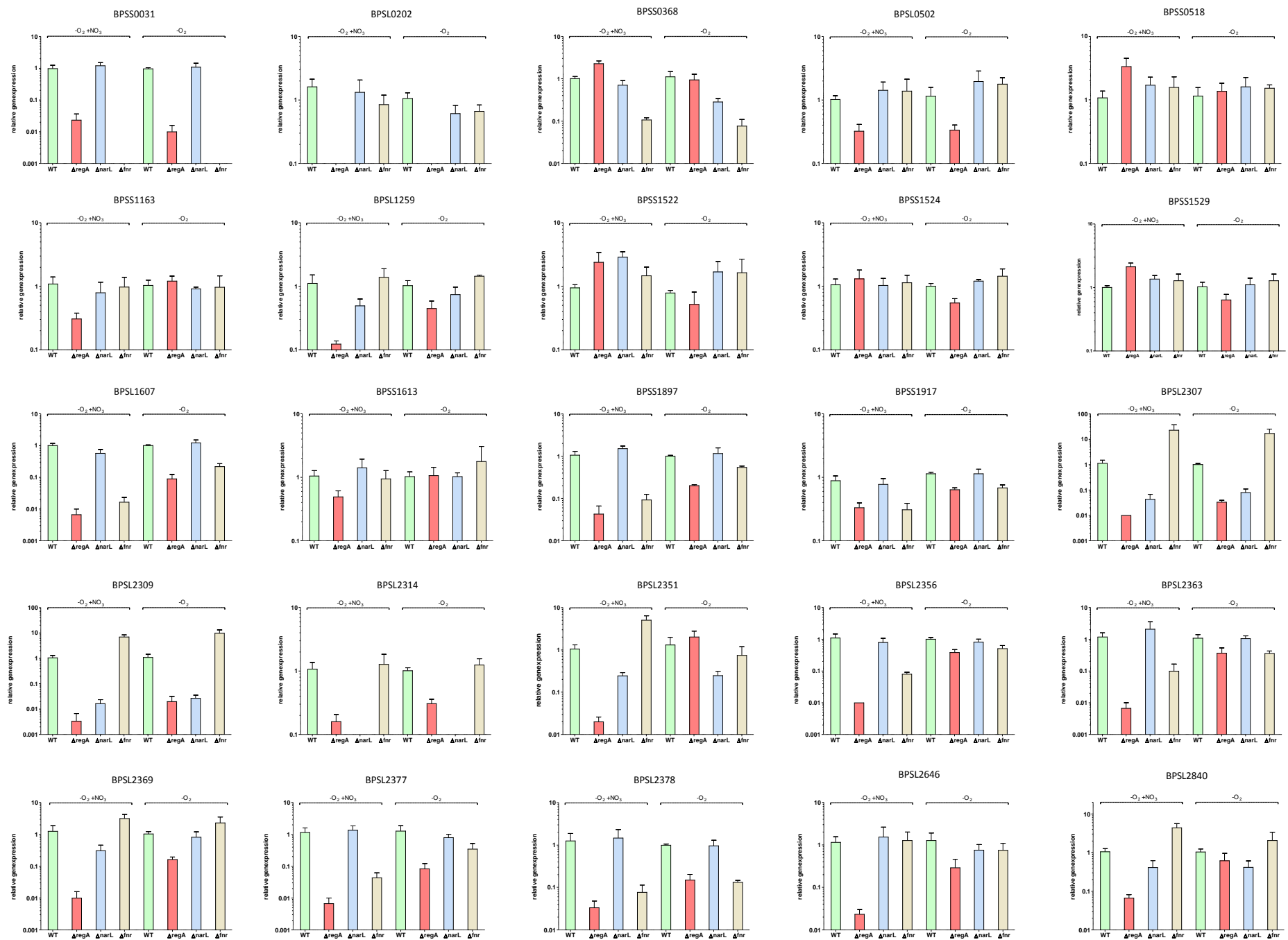

Supplement: S9 Fig — Data from qRT PCR experiments were normalized using the 23S rRNA gene as internal control. Mean values of three independent experiments are displayed. Error bars indicate standard error of the mean (SEM). BPSS0031—anaerobic growth regulatory protein FNR, BPSL0202—response regulator protein RegA, BPSS0368—TonB-like transport protein, BPSL0502—cytochrome d ubiquinol oxidase subunit I, BPSS0518—type VI secretion system secreted protein HcpI, BPSS1163—fumarate/nitrate reduction family regulatory protein, BPSL1259—putative cytochrome c oxidase subunit II related protein, BPSS1522—two-component response regulator, BPSS1524—intercellular spread protein, BPSS1529—membrane antigen, BPSL1607—nitrous-oxide reductase precurser, BPSS1613—type III secretion protein, BPSS1897—cytochrome o ubiquinol oxidase subunit II, BPSS1917—crp-family transcriptional regulator, BPSL2307—nitrite/nitrate transporter, BPSL2309—respiratory nitrate reductase alpha chain, BPSL2314—putative response regulator protein, BPSL2351—nitric oxide reductase subunit B, BPSL2356—anaerobic ribonucleoside triphosphate reductase, BPSL2363—putative 2-nitropropane dioxygenase, BPSL2369—cysteine desulfurase activator complex subunit SufB, BPSL2377—cation-binding hemerythrin HHE family protein, BPSL2378 3—ubiquinol oxidase polypeptide II precursor, BPSL2646—coproporphyrinogen III oxidase, BPSL2840 –flavohemoprotein Hmp. (PDF) [file ppat.1009604.s009.pdf]

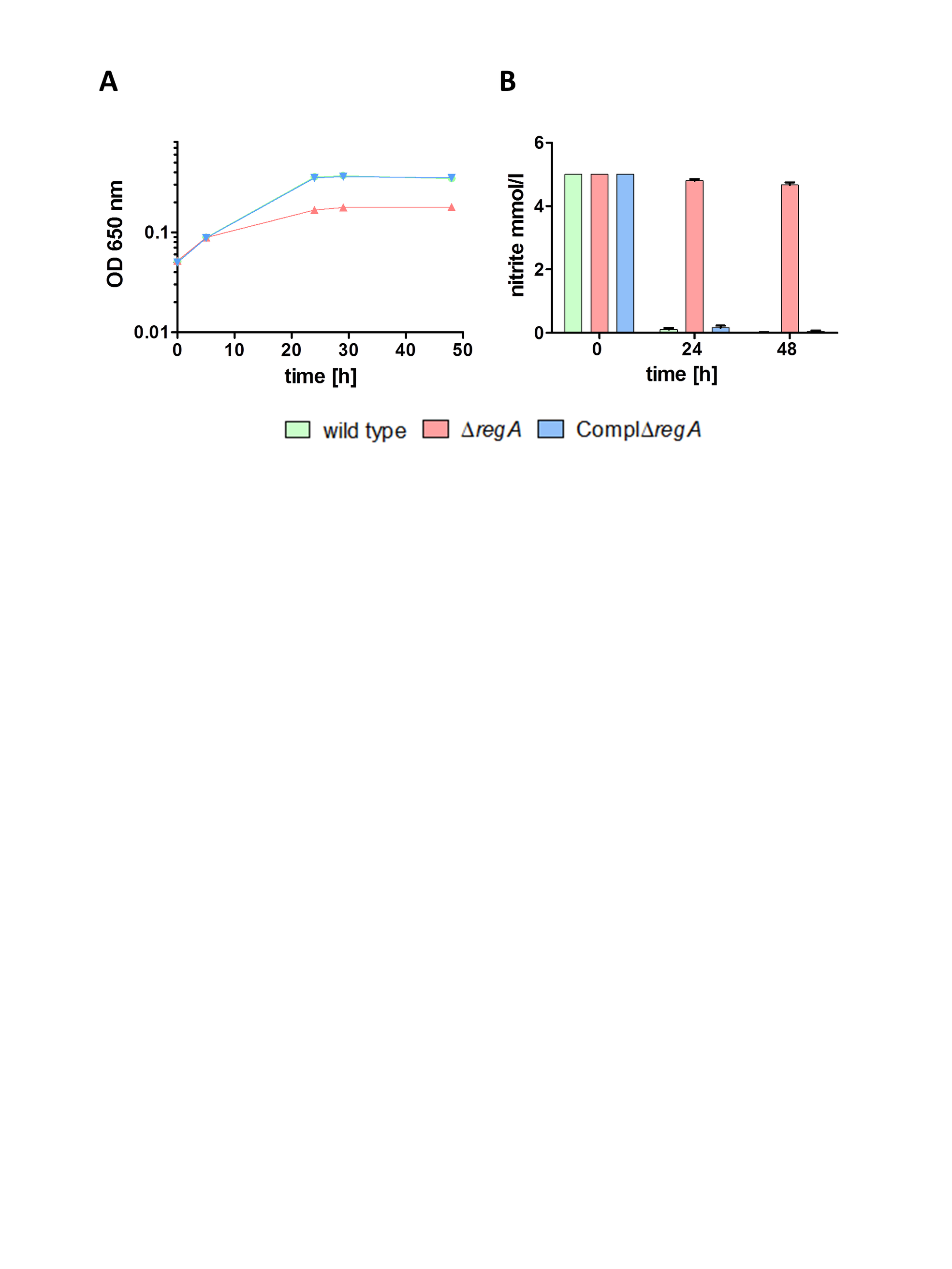

Supplement: S10 Fig — A: Cells were cultivated under anaerobic conditions in LB medium supplemented with 5 mM nitrite at 37°C and 140 rpm over a time period of 48 hours. Shown are mean values of three independent experiments. Error bars indicate standard error of the mean (SEM). B: Supernatants were harvested and concentrations of nitrite were measured as described in Material and Methods. Shown are mean values of three independent experiments. Error bars indicate standard error of the mean (SEM). (TIF) [file ppat.1009604.s010.tif]
